# Analytic validation and clinical utilization of the comprehensive genomic profiling test, GEM ExTra^®^

**DOI:** 10.18632/oncotarget.27945

**Published:** 2021-04-13

**Authors:** Tracey White, Szabolcs Szelinger, Janine LoBello, Amy King, Jessica Aldrich, Nathan Garinger, Matthew Halbert, Ryan F. Richholt, Stephen D. Mastrian, Cody Babb, Audrey A. Ozols, Laurie J. Goodman, Gargi D. Basu, Thomas Royce

**Affiliations:** ^1^Ashion Analytics, LLC, Phoenix, Arizona 85004, USA; ^*^These authors contributed equally to this work

**Keywords:** comprehensive genomic profiling, whole exome sequencing, RNA sequencing, precision medicine, pan-cancer

## Abstract

We developed and analytically validated a comprehensive genomic profiling (CGP) assay, GEM ExTra, for patients with advanced solid tumors that uses Next Generation Sequencing (NGS) to characterize whole exomes employing a paired tumor-normal subtraction methodology. The assay detects single nucleotide variants (SNV), indels, focal copy number alterations (CNA), TERT promoter region, as well as tumor mutation burden (TMB) and microsatellite instability (MSI) status. Additionally, the assay incorporates whole transcriptome sequencing of the tumor sample that allows for the detection of gene fusions and select special transcripts, including AR-V7, EGFR vIII, EGFRvIV, and MET exon 14 skipping events. The assay has a mean target coverage of 180X for the normal (germline) and 400X for tumor DNA including enhanced probe design to facilitate the sequencing of difficult regions. Proprietary bioinformatics, paired with comprehensive clinical curation results in reporting that defines clinically actionable, FDA-approved, and clinical trial drug options for the management of the patient’s cancer. GEM ExTra demonstrated analytic specificity (PPV) of > 99.9% and analytic sensitivity of 98.8%. Application of GEM ExTra to 1,435 patient samples revealed clinically actionable alterations in 83.9% of reports, including 31 (2.5%) where therapeutic recommendations were based on RNA fusion findings only.

## INTRODUCTION

Cancer has a high clinical burden and oncology therapies are expensive. It is estimated that 1,898,160 new cancer cases will be diagnosed and over 608,570 deaths are projected to occur in the United States in 2021 [1]. The prevalence of cancer is expected to rise over time, providing an expanding unmet need for genomic tests to help physicians treat patients in a more precise manner [2]. Identification of genomic alterations by Next Generation Sequencing (NGS) has become an efficient clinical tool, particularly for oncology as molecular markers can guide personalized treatment. However, the most broadly utilized tests are not comprehensive enough to cover all clinically relevant alterations for cancer therapeutic applications and are not adaptable to novel markers [3]. Precision medicine, using genomic and other molecular profiling technologies, to match a treatment to a patient’s specific tumor alteration(s), has been shown to improve survival and quality of life as well as economic outcomes versus single gene tests [4, 5]. However, tumor profiling is underutilized and only a proportion of targeted-therapy-eligible patients actually receive genomic tests that result in matched precision therapy [4, 6]. Additionally, the false-positive rate with tumor-only approaches is of special concern for patients of non-European background. A primary method for filtering out rare, benign variants of germline origin in tumor-only analysis is by comparison to public SNP databases [7]. However, these databases consist of SNPs that were assessed from pools of donors over-represented by individuals of European descent; thus, they are less effective for such filtering for genomes of non-European descent [8, 9]. The inclusion of somatic alterations in germline databases like ClinVar further impact sensitivity of tumor only approaches [10]. Furthermore, recent data suggest that filtering germline variants using population databases can overestimate TMB, as compared to germline subtraction approaches. False positive estimates have great implication in skewing ongoing clinical trial results and patient outcomes with respect to currently FDA approved immunotherapy drugs [11].

Currently available options for tumor profiling include immunohistochemistry (IHC), fluorescence *in situ* hybridization (FISH), and, more recently, small panel next-generation sequencing (NGS) [7, 12]. These options have a variety of limitations that vary by test, including subjectivity, low accuracy, and a propensity to miss certain clinically actionable variants [3]. NGS identifies single-nucleotide variants, insertions, deletions, copy number changes, and fusions that may be drivers of cancer growth. From a nationally representative sample of physicians in a recent study, three quarters of oncologists reported using NGS to guide treatment for patients with advanced treatment-refractory disease (34.0%), determine eligibility for clinical trials (29.1%), or decide on off-label use of FDA-approved therapies (17.5%) [13]. NGS is used in both laboratory-developed tests (LDTs) and FDA-approved oncology companion diagnostics (CDx), but there are limitations, including that NGS is restricted to hotspot panels, and not readily adaptable to include other genes identified during current or future drug development discoveries [14, 15]. Patient responses to targeted therapy can also be unpredictable, even when currently available NGS tests are used, due to resistance mechanisms or a variety of other phenomena that select hotspot panel tests may not be detecting [16]. Thus, a more comprehensive test, which covers all clinically relevant alterations in oncology is needed.

GEM ExTra uses DNA and RNA sequencing, and paired tumor/germline somatic identification to determine the frequency of mutations, fusions, and rare RNA variants in biopsied samples. Germline sequence subtraction is used to facilitate higher accuracy in detecting tumor specific alterations and has great implications for reporting TMB and MSI. This feature of GEM ExTra is especially important for the proper somatic variant calling for ethnic minority patients. Findings are mapped to a knowledgebase of FDA approved targeted treatment options as well as relevant clinical trial options. GEM ExTra has an ability to capture all documented, clinically relevant alterations while also allowing for new discoveries and to facilitate research. There are a few predominant laboratories that perform CGP tumor tests with the intended use of providing clinical decision support for therapy selection for cancer. GEM ExTra uses Whole Exome Sequencing (WES) for tumor DNA profiling, testing for all protein-coding genes in a sample, indicating that the test will be comprehensive now and in the future. GEM ExTra provides somatic variant calling based on tumor and matched germline sequencing allowing for improved discrimination of somatic variants from rare, benign germline variants when compared to tumor-only analysis used by other CGP tests. GEM ExTra also identifies clinically actionable transcript variants and fusion genes through transcriptome (RNA) sequencing. These are typically undetectable through conventional CGP tests, which only employ DNA analysis. The utility of GEM ExTra (19,396 genes + 169 introns) can be expected to supersede that of panel tests.

The GEM ExTra report provides physicians with a summary of key findings, focused on actionable variants where there is published scientific and medical literature in support of the finding, as well as potential clinical trial options. The test is designed to provide healthcare professionals with clinically actionable information to guide patient management decisions based on the genomic profile of a cancer patient’s tumor. This assay is an LDT, single-site assay performed at a CAP-accredited, CLIA-certified clinical genomics testing laboratory, Ashion Analytics, located in Phoenix, AZ.

## RESULTS

### Analytical validation

The GEM ExTra test was analytically validated by evaluating a variety of aspects covering the testing parameters including nucleic acid extraction and isolation, sequencing platform, and data analysis pipeline methodologies. The analytic performance characteristics of the assay were determined using a variety of tumor derived cell lines, and standards from commercially available sources commonly used to validate across multiple NGS platforms as well as clinical FFPE samples subjected to orthogonal testing against both low and higher throughput gold standard methods. Informatic cutoff filters were set at 5% allele frequency for non-hotspot variants, and 1% for hotspot mutations (Supplementary Table 1). Samples utilized for validation and the variant types detected are summarized in Table 1.

**Table 1:** Samples and variant types utilized in the analytic validation

Variants Validated	Validation Samples	Source of Validation	# of Variants for Validation
**SNV and Indels**	73 clinical samples, 4 reference standards	Tumor DNA	517 SNV events, 30 indel events
**Copy Number Alterations**	88 clinical samples, 2 reference standards	Tumor DNA	43 CNA events
**Gene Fusions**	32 clinical samples (FFPE), 2 cell lines, 4 commercial reference standards	Tumor RNA	91 fusion or special transcript events
**MSI**	29 patient samples orthogonally tested for MSI Status	Tumor DNA	Exome Wide
**TMB**	23 patient samples orthogonally tested for TMB status	Tumor DNA	Reported as low, intermediate, or high

### Assay performance quality metrics

Core Quality Metrics used in validation encompass pre-analytical, analytical, and post-analytical processes, and are detailed in Table 2. DNA input range was 50–1000 ng with a corresponding quality ratio of A260/280 of 1.8 to 2.0. Depth of sequencing coverage was minimally 240x for tumor and 100x for normal samples. The RNA input quantity was determined to be in the range of 25–1000 ng based on a ≥ 20% DV200 value. Total RNA sequencing reads were > 100 million.

**Table 2:** Assay performance quality metrics

Metric	Details
RNA Quantity	25–1000 ng input based on DV200 value
RNA Quality	≥20% DV200
DNA Quantity	50–1000 ng input
DNA Quality	260/280 1.8–2.0
Library Quantification	~300 bp
Onboard Q30	≥80%
PhiX	~0.5–1.0
Depth of Coverage (DNA)	Minimum 240X tumor, 100X normal
Uniformity of Coverage (DNA)	≥90% at 40x
Total Reads (RNA)	≥100 million
Percent Aligned (RNA)	≥50%

### Overall performance

Patient samples with a representative distribution of both tumor sample types (~75% FFPE samples and the remaining ~25% were fresh-frozen, cell pellets, or bone marrow aspirates) were chosen for method comparison. FFPE samples ranged in age from > 4 years to < 1-year-old. Tumor content of samples ranged from 30–95%. Overall, 183 patient samples from 132 tumor types were used in the validations. The overall performance of the assay is outlined in Table 3.

**Table 3:** Overall performance of GEM ExTra

	Variant	Specification
**Analytic Sensitivity**	Single Nucleotide Variants (MAF > 1%)	99.6% (CI 98.8–99.9)
	Small Insertions & Deletions (MAF > 2%)	96.8% (CI 85.9–99.7)
	Copy Number Alterations	97.7% (CI 89.6–99.7)
	Gene Fusions	93.1% (CI 86.3–97.1)
**Analytic Specificity (PPV)**	Specificity	>99% (CI 97.4–99.2)
**MSI**	Exome Wide	>99.9% Concordance
**TMB**	Exome wide	91% Concordance

### Sensitivity and specificity of single nucleotide variants (SNVs) and indels

Somatic SNVs and short indels are identified by standard freebayes filters that calls short haplotype sequences within both normal and tumor alignments. The frequency of the alternative haplotype relative to the reference haplotype is calculated for both the tumor sample and for the normal sample. After filtering for quality and allelic frequency a 2x2 contingency table is constructed with each haplotype count in the normal and tumor. A one-sided, Fisher’s exact test is performed, and FDR of 1% is established with Benjamin and Hochberg to identify the final call set.

**Figure 1:**
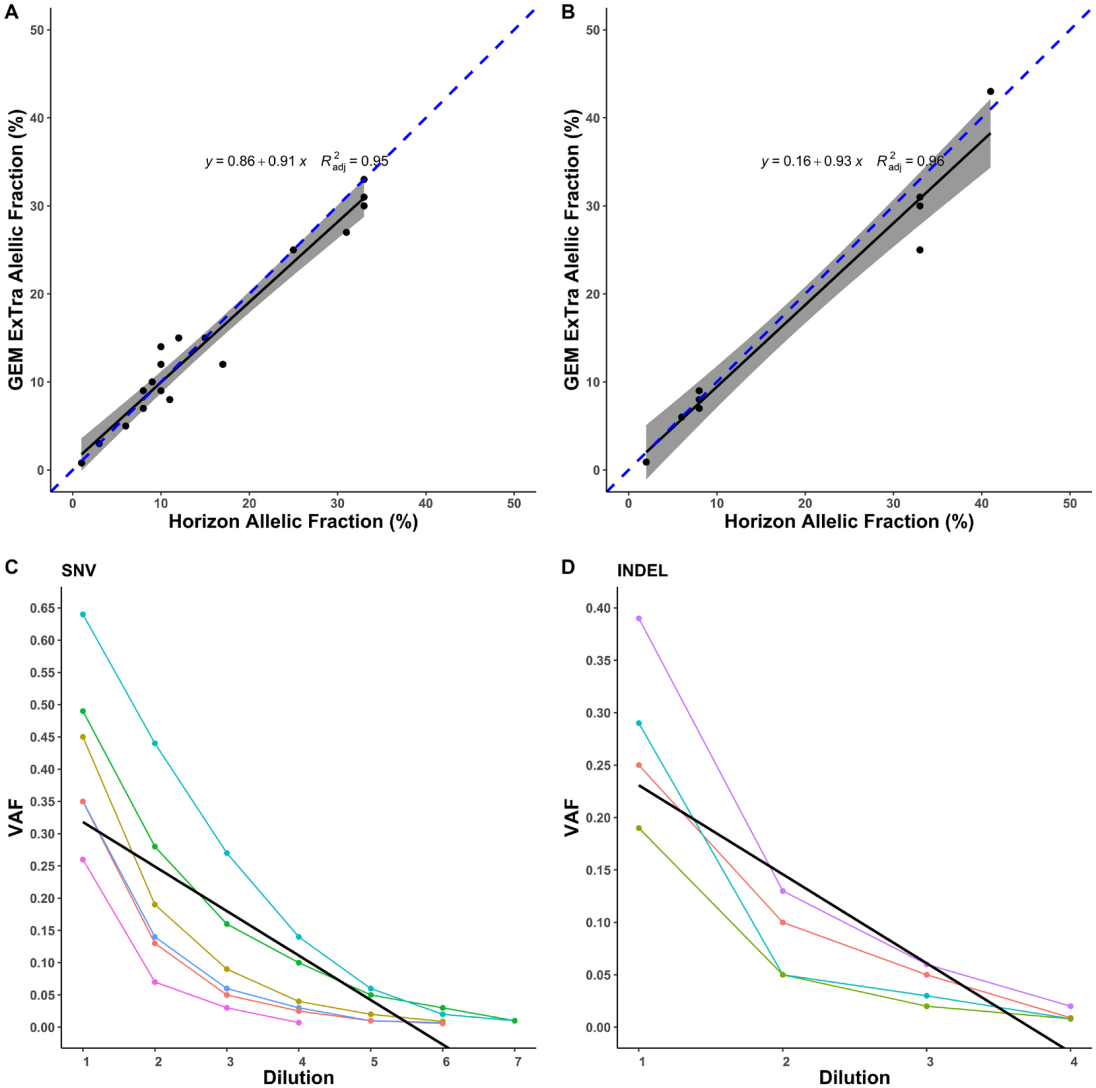
Performance of SNV and Indel detection by GEM ExTra. (**A**) Correlation of GEM ExTra SNV VAF to Horizon reference standard. (**B**) Correlation of GEM ExTra Indel VAF to Horizon reference standard. The black line is the regression line, and the gray area is 95% confidence interval. The dashed blue line indicates x = y. (**C**) Serial 1:1 dilution of 6 SNVs and their corresponding VAFs. Black line indicates the linear regression of the data. (**D**) Serial 1:1 dilution of 4 Indels and their corresponding VAFs. Black line indicates the linear regression of the data.

Horizon’s Quantitative Multiplex reference FFPE DNA includes SNVs and indels with validated allele frequencies. This sample has 28 confirmed variants within our reportable range. Correlation of the expected and observed allelic frequency measurements by GEM ExTra showed high concordance, *r*^2^ = 0.95 for SNVs and *r*^2^ = 0.96 for indels (Figure 1A and 1B). In addition, we found sensitivity of 92.8% (26/28), with two discordant variants detected by the system that were below the established bioinformatics pipeline threshold of GEM ExTra to be called, and thus were filtered out. We also performed accuracy studies of SNVs and indels using patient samples. A total of 159 mutations were selected from 80 genes. 148 were tested by orthogonal NGS method, 6 by IHC, and 5 by PCR based method. There were 51 mutations where either GEM ExTra or orthogonal testing lab did not provide an allelic fraction estimate (GEM ExTra = 1, Orthogonal Lab = 50). There was an agreement of 99.5% in the calls between the methods utilized.

### Sensitivity and specificity of copy number alterations

Copy number is determined based on coverage difference between the “normal” and the “tumor” specimen determined on a logarithmic scale. More specifically, as FFPE samples are inherently more noisy, prior copy number calling alignments are evaluated and normalized for insert size, GC content and dinucleotide bias. Similar insert size distributions minimize alignment bias between samples, dinucleotide correction allows for controlling fragmentation bias between FFPE preparations, and GC correction controls for differences in capture efficiency. Normalized alignments are quantified and a log2 ratio of tumor counts compared to normal counts is calculated for each genomic region. Finally, for each gene, log2 ratio difference is calculated in addition to log2 ratio of the gene compared to chromosome arm to account for ploidy. CNV specificity was established using patient samples called by GEM ExTra as compared to an orthogonal method. A total of 31 copy number alterations in 10 genes were assessed (Focal Deletion = 5, Focal Gain = 26) by NGS, FISH and IHC and 100% of these were concordant.

### Limit of detection studies

To establish Limit of Detection (LOD) for the test to detect a DNA variant in a background of assay-relevant biological matrix, studies were conducted to demonstrate a putative LOD for each variant type. A dilution series was conducted to identify the lowest reliable mutant fraction. LOD was assessed for 10 unique tumor/germline sample pairs where the tumor contains one of 10 mutations (SNV = 6, Indel = 4) with evidence of clinical significance. 5/6 SNVs were hotspot, and 1/4 indels was a hotspot alteration. Serial dilutions were generated 1:1 from original tumor and germline samples down to 1:64. SNVs and Indels were consistently detected down to 1% VAF (Figure 1C and 1D). Manual inspection of the data showed that at the highest dilution (1:64) all SNVs were called by our pipeline, however, 4 of them were filtered out after setting variant call detection to 1%. Indels were detected down to 1:8 dilution with one indel at 2% and the other 3 indels detected just below 1% VAF. All the hotspot mutations were detected below 1% VAF, and thus we set LOD for hotspot at 1%, and were more conservative for non-hotspot at LOD of 5%. To confirm our studies, all mutations were visually inspected using Integrative Genomics Viewer (IGV) [17].

### MSI studies

We estimate microsatellite instability by scanning the tumor-specific indels for mono-, di-, or tri-nucleotide repeats. Those with a length greater than or equal to three are tallied. Above a cutoff of six across the exome, the sample is declared microsatellite instable high (MSI-H), otherwise it is labeled as microsatellite stable (MSI-S).

To validate the MSI test we performed accuracy studies using 29 patient samples tested by an orthogonal, PCR-based approach. Patient samples were selected to demonstrate a range of tumor content from 20% up to 90% by tumor estimate. MSI status ranges from stable (*n* = 19) to high (*n* = 10). All MSI-H samples by GEM ExTra were classified as MSI-H by the orthogonal PCR assay with concordance of > 99.9%.

**Figure 2:**
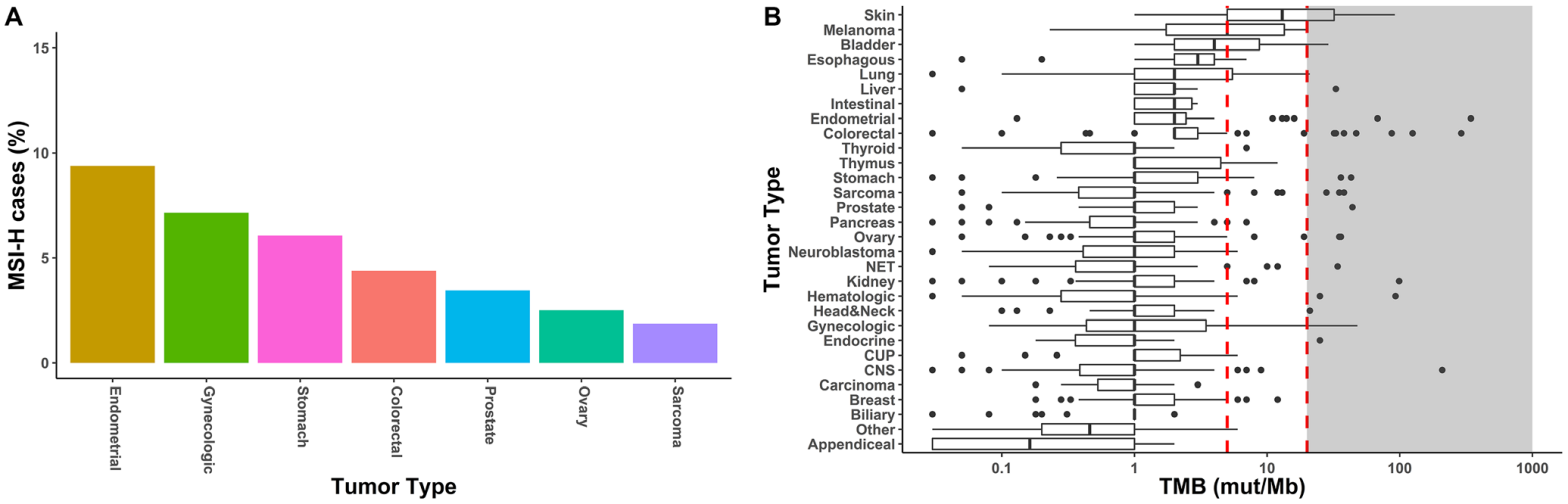
Biomarkers for immunotherapy by GEM ExTra. (**A**). Frequency of high microsatellite instability in GEM ExTra. 7/30 tumor types harbored MSI-H tumors. (**B**) TMB landscape in GEM ExTra. Boxplots show distribution of TMB scores in mutation/Megabase. Black line within boxes shows median TMB score, the right edge of the box is the 75th percentile of interquartile of TMB scores, left edge of box is 25th percentile interquartile of TMB scores. Red, dashed lines indicate TMB threshold of 5 mut/MB for low TMB, and 20 mut/Mb for high TMB. Black dots or outliers are TMB scores outside the 1.5 interquartile range. Shaded are indicates TMB >= 20 mut/Mb.

We estimated the frequency of microsatellite instability in our clinical sample cohort of 1,499 samples across, approximately 30 different tumor types. Compiled tumor types were based on SNOMED code, as well as similar tumor origin or histology and combined into a single summary Disease group (e.g., Pancreas Tumor Type contained all samples classified as Carcinoma of pancreas or Carcinoma of ampulla of Vater by SNOMED but did not contain samples with Carcinoma of endocrine pancreas SNOMED classification). GEM ExTra identified approximately 1.2% of clinical cases (*n* = 18) as having MSI-H status. Tumor types with the highest frequency of microsatellite instability were endometrial (9.4%), gynecologic (7.1%), stomach (6.1%), colorectal (4.4%), prostate (3.5%), ovarian (2.5%), and sarcomas (1.9%). Although percentages are lower compared to previous pan-cancer MSI studies, general trends correlate with preponderance of MSI-H cases in gastrointestinal and cancer of the reproductive organs [18] (Figure 2A).

### Tumor mutation burden (TMB) studies

TMB is calculated as the number of coding somatic alterations per million base pairs of target space in GEM ExTra. TMB range of <5 mutations/MB of DNA is considered “low”, a range of 6< mut/Mb to <19 mut/MB is considered intermediate, and a >20 mut/Mb is considered “high”. These ranges of TMB were based on extensive literature review, correlation studies with MSI status in select cancer types, and clinical trial enrollment criteria. TMB was correlated with 22 patient samples analyzed by an externally validated NGS method. Since methodologies (the external method was a tumor-only, large panel,) and thresholds (which were not disclosed by the external laboratory) differed between the two assays, concordance was assessed as classification into low, intermediate, and high results. In terms of the classification into “Low”, “Intermediate”, and “High” categories, there was a 91% concordance between the methods (data not shown). Two of the 22 results were discordant and may be due to differences of thresholds between the two assays.

Review of the 1,509 GEM ExTra clinical samples demonstrated a similar TMB distribution compared to other large scale sequencing studies (Figure 2B). Median TMB ranged between 13 mut/Mb for skin tumors and 0.2 mut/Mb for appendiceal tumors. As previously reported, tumors with the common disease mechanism of high mutagenic burden such as melanoma (5 mut/Mb), bladder (4 mut/Mb) and lung (2 mut/Mb) were also among the higher mutationally burdened tumor types [19]. Overall, we found 42/1,509 (2.8%) of tumors were classified as TMB-High, and the tumor types with the most frequent TMB-High classification were skin (41.4%), gynecologic (14.3%), and melanoma (11.1%) (data not shown). Eighteen of 30 tumor types harbored at least 1 sample with high TMB. However, we found tumors of the appendix and gallbladder associated with low TMB only, with some studies suggesting low mutagenic burden for these tumor types [20, 21].

### RNA standard reference comparison and patient tumor sample orthogonal testing

A variety of cell lines, and universal reference material was utilized to determine accuracy with respect to fusion calls. A total of 62 events were evaluated, with a demonstrated sensitivity of 91.2%, a specificity of 100%, and a PPV of 100%. AR-V7, MET exon 14 skipping, and EGFRvIII variants were all accurately called in the reference samples.

To assess and compare RNA sequencing results between Ashion and external laboratory testing, 31 individual patient tumor samples were tested using external laboratory methods (NGS, FISH). Fifteen different tissue types (both positive and negative patient samples) were included. We saw no correlation between age of sample, and quality of analyte or sequencing. 100% agreement of results was achieved (Supplementary Table 2).

### Precision

To determine whether the assay returns the same result regardless of minor variations in testing conditions which can introduce random error, 21 samples were evaluated. Samples were selected based on known clinically significant mutations with a range of variant allele frequencies as well as the associated target tissue to include challenging specimens. Tumor types included astrocytoma, colon, GBM, GIST, lung, lymphoma, melanoma, neuroblastoma, ovarian, pancreas, sarcoma, stomach and urothelial. Minimum inputs were used for each replicate (50 ng DNA and RNA input based on DV200 score [22]). Correlation acceptability was set at an average >90% agreement of all (repeatability and reproducibility) replicates.

Within-run replicates of 21 patient samples were tested in triplicate from separate aliquots of DNA/RNA on the same run and flow cell, demonstrating the repeatability of the assay. To determine whether the assay was reproducible, between-run replicates of 21 patient samples were tested by different operators from separate aliquots of DNA/RNA on different days across different instruments and lot numbers (where available). Observed mutations were reported and assessed for precision. The precision of variants of clinical significance was 100% agreement in the calls within the informatic cutoffs utilized for hotpot and not hotspot alterations (data not shown).

### Clinical utilization

To estimate the clinical utilization of the GEM ExTra assay, the detection rate of clinically actionable alterations was calculated from the clinical reports generated between April 2018 and December of 2019. In the GEM ExTra assay, multiple somatic alteration types are reported including “Clinically Actionable”, “Additional Significant Alterations”, and “Variants of Unknown Significance.” Clinically Actionable alterations are defined as alterations that are associated with on-, or off-label FDA approved drugs or clinical trial enrollment for a specific somatic alteration identified in a patient’s tumor. Additional Significant Alterations are somatic changes with published evidence for diagnosis or prognosis in patient’s disease. Variants of Unknown Significance are alterations that are not predictive of response or resistance to targeted therapy based on scientific evidence. Below we summarize the reported clinically actionable alterations.

**Figure 3:**
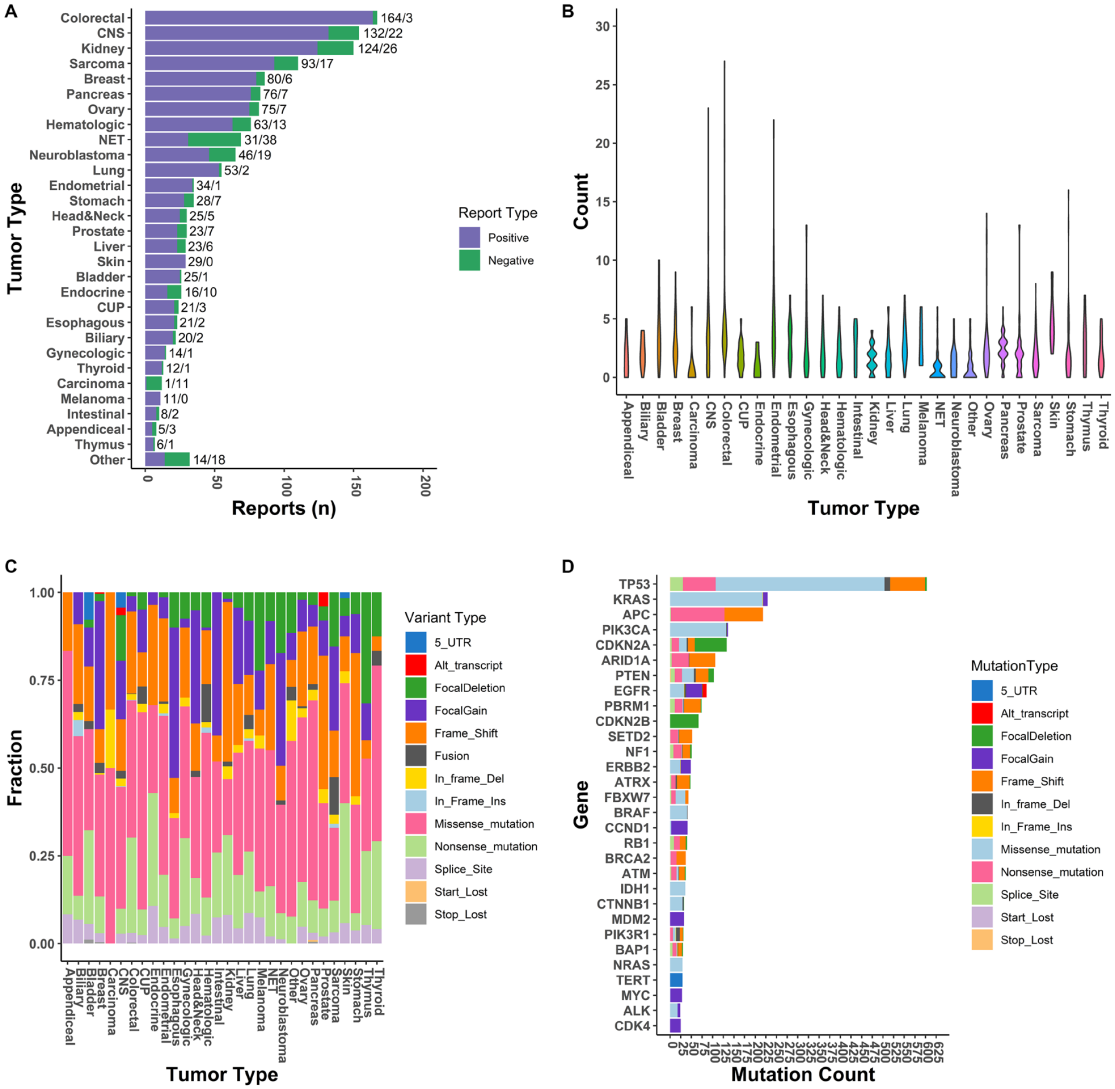
Performance characteristic of GEM ExTra assay. (**A**) Tumor specific positivity of GEM ExTra. (**B**) Violin plot of the number of clinically actionable events reported per tumor type. (**C**) Frequency of variant types across tumor types. (**D**) The thirty most common clinically actionable genes and number of variant types detected in each gene.

A total of 1,509 clinical reports were generated during this two-year period (2018 = 369, 2019 = 1140) for a total of 1435 individual patients. Overall, 83.9% of reports (*n* = 1261) included both tumor DNA and RNA profiling, while 17% (*n* = 248) were tumor DNA profiling only (Supplementary Table 3). The distribution of positive and negative (clinically actionable alternations) reports for each tumor type is listed in Figure 3A. The most predominate tumor types assayed were colorectal (11.0% of total tested), CNS (10.2% of total tested), and kidney (9.9% of total tested).

We found that 83.9% of tumor samples harbored at least one clinically actionable alteration (defined as positive) and the rest defined as negative, with a total of 1267 positive and 242 negative reports (Detection Rate: 2018 = 76.4%, 2019 = 86.4%). Overall, 3535 clinically actionable mutations were identified in our cohort (1864 unique mutations), with a median of 2 clinically actionable alterations per tumor (mean = 2.93 ± 2.37) showing extensive variation across cancer types (Figure 3B). Tumors with highest number of actionable mutations included skin (4.9 ± 2.2), endometrial (4.5 ± 3.9), and colorectal (4.1 ± 3.5). These results generally agree with previous estimates of driver events per patient in these tumor types [23]. This is somewhat lower than previously reported in a pan-cancer study (4.6/tumor) of whole genomes, which also included driver copy number alterations which are not called out as actionable with GEM ExTra [24]. Mean coding SNVs (i.e., missense, nonsense, stop codon) was 1.9 ± 1.4 per tumor which is within the range of predicted driver mutations in cancer [25].

Inspection of mutation profiles in our cancer cohort showed expected driver events in several tumor types (Figure 3C). For example, approximately 45% of driver events in esophageal cancer included focal amplifications in cell cycle genes such as CCND1/2/3 and CDK4/6/9, in addition to amplifications in ERBB2, KRAS, MYC [26]. RNA fusions are significant contributors to tumorigenesis in sarcoma and hematologic malignancies, and these alterations were most common (>10%) in these tumor types. Alternative transcripts were recurrently identified in EGFR (i.e., vIII, vIVb) within CNS tumors in AR (e.g., v7) within prostate tumors, and in MET (e.g., exon 14 skipping) within breast and lung (data not shown) tumors. Finally, point mutations in BRAF/NRAS/PTEN/TP53 are key driver events in Thyroid cancer, and were identified in approximately 80% of thyroid tumors in this study [27, 28].

**Figure 4:**
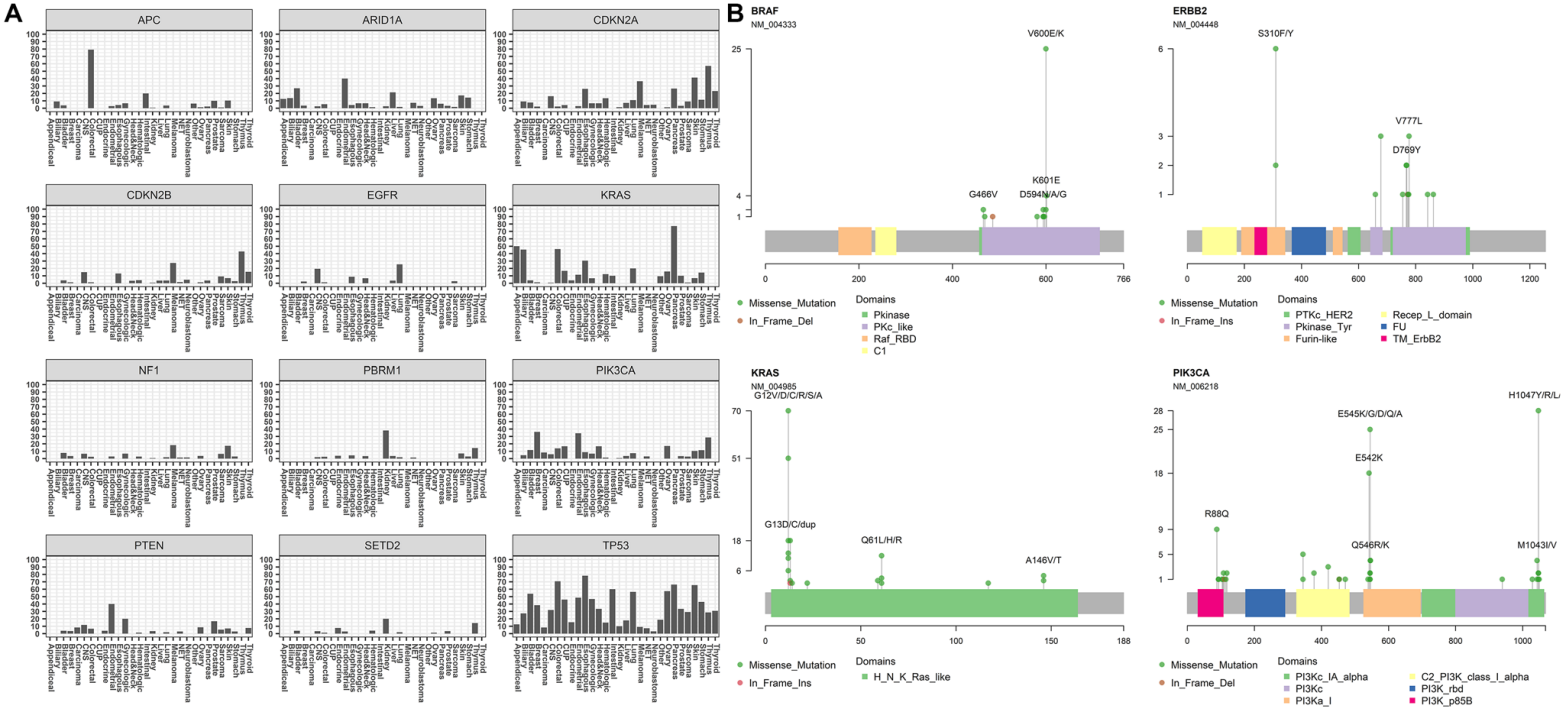
Mutation profiles of clinically actionable genes in GEM ExTra. (**A**) The twelve most reported genes and their mutation distribution across tumor types. (**B**) Four selected clinically relevant genes with hotspot mutations and their frequency among various tumor types. Alterations colored by mutation types and their position with respect to domain structure is shown. RefSeq gene ID and count frequency shown on y axis.

Overall, the most frequently mutated gene was TP53 with 603 clinically actionable alterations reported, with 66% of the tumor-specific mutations being hotspot or recurrent missense mutations (Figure 3D). The GEM ExTra assay also identified a similar driver mutation profile in KRAS as reported by TCGA. For example, 95% of KRAS alterations were missense oncogenic alterations primarily in hotspot codons such as G12, G13, Q61, Q22, A59, K117, and A146 while 5% of the tumors harbored KRAS amplifications, primarily identified in esophageal cancer. In fact, 80% of KRAS alterations in esophageal tumors were amplifications, which correlated with the TCGA dataset [29]. Thirty percent of tumors harbored at least one clinically actionable TP53 alteration, with the highest frequency in esophageal tumors (80%) and this correlates well with previously reported studies [30, 31] (Figure 4A). In addition, GEM ExTra identified KRAS alterations in approximately 77% of pancreatic tumors which generally correlates with previous estimates in this tumor type [32, 33]. Pancreatic tumors with KRAS actionable alterations were mainly driven by G12 codon alterations. Of the 65 KRAS-mutant tumors 57 (87.6%) harbored a G12A/C/D/R/S/V mutation. Although the tumor samples received for sequencing encompass a wide spectrum of pre-, and post-treatment primary and metastatic tumors with complex histopathology, GEM ExTra assay findings generally correlated with previously reported driver gene frequencies including but not limited to CDKN2A alterations in melanoma [34], EGFR in lung and CNS tumors [35], PIK3CA hotspot in breast and endometrial tumors [36], and PTEN loss of function alterations in endometrial tumors [37], suggesting the clinical utility of the GEM ExTra assay in the detection and reporting of clinically relevant somatic alterations in a wide spectrum of sample types. Hotspot alterations in clinically relevant cancer drivers were consistently identified across cancer types suggesting their pan-cancer significance [38, 39] (Figure 4B).

**Figure 5:**
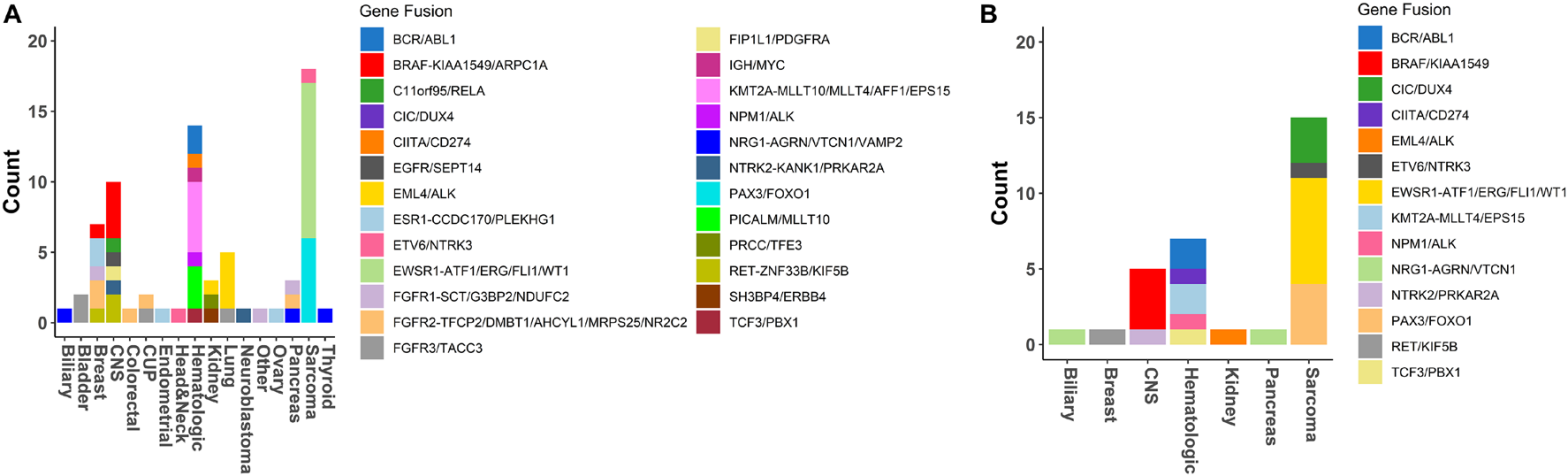
Fusions detection in GEM ExTra. (**A**) Fusions detected by tumor type. (**B**) Fusion’s detected in tumors types with RNA only findings. For those main fusion genes (e.g., BRAF) that were found to be fused with multiple partner genes (e.g., KIAA1549, or ARPC1A) the partner genes are separated from main fusion gene by a dashed line “-”, and the other partners listed consecutively, separated by a forward slash “/”.

In this study 75 clinically actionable RNA fusions were identified among all samples where RNA quality was sufficient for sequencing (75/1261), an approximate 5.9% detection rate across our pan-cancer cohort. Overall, clinically actionable RNA fusions were most frequent in sarcomas (18.2%) and hematologic malignancies (18.9%). Among the 75 reports with clinically actionable RNA fusions, 31 had RNA findings only. Therefore RNA sequencing and fusion detection provided and increased yield in 31/1261 reports (2.5%). Of the remaining 44 samples, 41 harbored RNA fusions, which were also supported by a related structural alteration in the tumor DNA in the form of a translocation, inversion, deletion, or duplication. Thus, approximately half of the fusions (54.7%) were supported by genomic rearrangements. We found that hematologic, sarcoma and lung tumors harbored the highest fraction of clinically actionable fusions (Figure 5A and 5B). Additionally, in 75% (15/20) of sarcoma cases, where an actionable RNA fusion was detected, the sole alteration was identified in the RNA suggesting the importance of tumor RNA profiling in the tumor type. As expected, lung tumors with driver fusions harbored mostly EML4/ALK fusions, sarcomas were driven mostly by EWSR1-related and PAX3/FOXO1 fusions, while hematologic malignancies with a spectrum of BCR/ABL1, KMT2A-related, and IGH/MYC fusions. We also identified recurrent KIAA1549/BRAF fusions in Pilomyxoid astrocytoma tumors which is an emerging diagnostic and prognostic marker in pediatric low-grade gliomas that predicts positive response to certain MEK inhibitors [40, 41]. Finally, several lung and breast cancer cases have been identified harboring MET exon 14 skipping detected by GEM ExTra tumor RNA sequencing, suggesting FDA-approved therapeutic options such as MET-inhibitors in these tumor types as recommended by NCCN (data not shown).

## DISCUSSION

Novel targeted therapies and immunotherapies are now providing patients with increased survival in various cancer types. NGS-based testing to guide therapeutic decisions is commercially available from many different diagnostic laboratories, and NGS brings an ability for physicians to save time and tissue samples, while identifying approved therapies, appropriate clinical trials, or rare, actionable mutations [42, 43]. Although clinically useful, existing fixed-panel NGS assays for tumor profiling are not truly comprehensive, as they are only limited to genomic alterations that are known to be clinically relevant at the time of their design. As new relevant markers are discovered, these tests will become outdated, and thus patients will not receive all the information that could beneficially inform their care, due to the lag built in by the need to develop and analytically validate up-to-date test panels. Furthermore, recently commercialized WES/RNA sequencing tests lack short turnaround times to provide superior care to cancer patients [15, 44, 45]. And, while most of these newer and more comprehensive tests employ tumor/germline subtraction, they lack the sequencing coverage of the GEM ExTra test, and therefore may suffer from lower accuracy and sensitivity to detect rare fusions and transcript variants.

Recent studies have shown that calculations of TMB using tumor-only assays may be falsely elevated compared to those determined by germline subtraction [11], as the GEM ExTra assay employs. This likely accounts for the two discrepant categorizations (low versus intermediate) between the GEM ExTra and external methods. TMB has increasingly been studied in different tumor types to identify patients who will benefit from immunotherapy, which is becoming standard of care therapy in several cancers. Recently, the FDA granted accelerated approval to pembrolizumab for the treatment of adult and pediatric patients with unresectable or metastatic solid tumor with TMB (≥10 mut/Mb), as determined by an FDA-approved test, that have progressed following prior treatment and who have no satisfactory alternative treatment options. Although there are limitations with TMB analysis, including that the standards for determination and reporting are currently not well established, the test has potential to make cancer treatment more precise [46].

We developed and analytically validated a comprehensive genomic profiling assay with a 14-day turnaround time, that can be adapted to all future tumor profiling needs due to combined DNA and RNA analysis. The GEM ExTra assay not only uses WES for tumor DNA profiling, but also identifies clinically actionable transcript variants and fusion genes through RNA sequencing, both of which ensure that GEM ExTra will be comprehensive in the future. GEM ExTra reports on more clinically actionable genes than other leading FDA approved CGP tests, which use fixed targeted panels and includes copy number events, MSI, and TMB, providing a holistic picture of actionable DNA-associated mutations. Moreover, the test employs tumor-normal somatic identification to determine tumor specific alterations as well as assessing TMB by the most accurate methodology.

In this study, the analytic performance characteristics of the assay were validated by comparison of patient samples to reference assays, and actionable variants were identified in tumors to guide oncology patient management decisions. The test was utilized in over 1400 patient samples during a period of April 2018 and December 2019 across cancer centers to detect multiple actionable alterations in a variety of cancer types. Reports of these actionable mutations were utilized to inform patient care, including matching patients to available targeted therapies or clinical trials. The data from the clinical laboratory testing is generally concordant with data in the literature and emphasizes the value of the use of this pan-cancer comprehensive genomic test for the clinical management of patients with advanced cancer. As of December 2019, Ashion was added to the list of commercial laboratories that are designated to identify and refer eligible patients to the NCI-MATCH trial [47].

## MATERIALS AND METHODS

### Reference materials

Studies were performed using both thoroughly characterized, commercially available reference materials, as well as patient samples tested by validated methods.

In the DNA accuracy study, matrix-specific samples were used when available. Horizon’s Quantitative Multiplex (Horizon Dx) - reference FFPE DNA including SNVs and indels with validated allele frequencies. This sample had 28 confirmed variants within our reportable range.

The following characterized samples were included in the RNA accuracy study. Matrix-specific (FFPE) samples were used when available.

- 22Rv1 cell line (Sigma Aldrich) – well established prostate cell line that expresses high levels of the ARv7 variant, which is known to confer resistance to AR-targeted therapies. ARv7 positive patients also have a shorter overall survival.
- MCF-7 cell line (Sigma Aldrich) – well established breast cancer cell line containing several confirmed fusions.
- Universal Human Reference (UHRR) (Agilent) – commercial standard pool of 10 cancer cell lines with known fusions.
- SeraSeq Fusion RNA v2 (SeraSeq) – commercial FFPE reference material containing known fusions as well as EGFR vIII and MET exon 14 skipping events. EGFR vIII mutation is seen in glioblastoma multifoma, breast cancer, and head and neck squamous cell carcinoma, and is relatively resistant to treatment with conventional anti-EGFR treatments. MET exon 14 skipping events are seen in lung cancer and are known to respond to MET/ALK inhibitors.
- SeraSeq Myeloid Fusion Mix (SeraSeq) – commercial fusion reference material.
- Horizon Dx 5 Fusion Multiplex (Horizon Dx) – commercial FFPE fusion reference material.

### Tissue specimens

Tumor tissue was evaluated by a pathologist for neoplastic content and macrodissected when necessary.

### Nucleic acid isolation

Tumor genomic DNA was extracted from formalin fixed paraffin embedded (FFPE) tissue per Qiagen AllPrep DNA/RNA FFPE Kit protocol using QIAcube automation (Qiagen). Fresh frozen tissue was extracted per protocol using the Qiagen AllPrep DNA/RNA Mini Kit (Qiagen). DNA is extracted from peripheral blood or saliva per Qiagen DNA Blood Mini Kit using QIAcube automation (Qiagen). Established quality control metrics were used to evaluate DNA quality (260/280). Analyte may be stored at ≤ –80^°^C if not proceeding directly to library construction. DNA was sheared per protocol and a quality control check performed.

### Library prep

DNA libraries were prepared using the KAPA HyperPrep library kit (Roche). The process includes end repair and A-tailing, which produces end-repaired, 5′-phosphorylated, 3′-dA-tailed dsDNA fragments; adapter ligation, during which dsDNA adapters with 3′-dTMP overhangs are ligated to 3′-dA-tailed molecules, followed by library amplification. A quality control check is performed for size (fragments should be ~300 bp) and yield (a minimum of 500 ng). Libraries may be stored at ≤ –20^°^C if not proceeding directly into the capture process per manufacturer’s specifications.

RNA libraries were prepared using the KAPA RNA HyperPrep with Riboerase kit (Roche) for Total RNA sequencing. The process includes depletion of rRNA by hybridization to complementary DNA oligonucleotides; fragmentation using heat and magnesium; 1st strand cDNA synthesis using random priming; combined 2nd strand synthesis and A-tailing followed by library amplification. A quality control check is performed for size (fragments should be ~300 bp) and concentration (a minimum of 1 ng/μL). Libraries may be stored at ≤ –20^o^C if not proceeding directly to sequencing per manufacturer’s specifications.

### Sequencing

Targeted sequences from DNA libraries were captured using a custom IDT xGen exome capture (Integrated DNA Technologies) probe set that targets coding regions of 19,396 genes as well as 169 introns relevant to oncology. The capture provides for double coverage of 440 genes relevant to oncology (see Appendix A for complete list). These specific genomic regions are captured using the IDT xGen Universal Blockers -TS Mix and IDT xGen Lockdown Probes for Illumina sequencing platform. The xGen Universal Blockers bind to ligated sequencing adapters present within the library molecules reducing nonspecific binding of adapter arms. Individually synthesized, 5′–biotinylated Lockdown Probes are bound to the targeted genomic regions of interest. The captured targeted regions are amplified, and a quality control check is performed for size (fragments should be ~300 bp) and concentration (a minimum of 6 ng/μL). Captures may be stored at ≤ –20^°^C if not proceeding directly to sequencing per manufacturer’s specifications.

DNA and RNA samples were pooled and sequenced using Illumina NovSeq 6000 Sequencing Instruments and reagents and PhiX Control (Illumina). Sequence data is processed using a customized analysis pipeline (both publicly available and Ashion proprietary tools).

### Data analysis pipeline

Once a sequencing run is complete, bioinformatics analysis is triggered by the Ashion Clinical Laboratory Information System (ACLIS). Using a queuing system, Illumina BCL files are converted to FASTQ files (raw sequence) and aligned to the genome, using BWA-MEM.

In the DNA workflow, PCR duplicates are marked with Samblaster and sorted genomically with Sambamba to create a final BAM file. Tumor point mutations are detected with Freebayes. Structural variants are detected with Manta. Amplifications and deletions are detected with a custom perl script, as are microsatellite instability and tumor mutational burden (TMB).

The RNA workflow consists of fusion detection with STAR-Fusion, followed by variant filtering with FusionInspector (part of the Trinity Cancer Transcriptome Analysis Toolkit).

Each of these variant callers produces a VCF file which can then be inserted into the ashionMarkers01 variant database. FASTQ files, VCF files, and BAM files are then packaged, encrypted and copied to a permanent storage area.

The pipeline framework is built with GNU Bash and uses the file system to detect and mark what steps are completed or needs to run next. ACLIS creates the files necessary to kick off the processing for various cron jobs which listen to initiate data processing.

## SUPPLEMENTARY MATERIALS





## Abbreviations

CDx: Companion Diagnostics
CGP: Comprehensive Genomic Profiling
CNA: Copy Number Alterations
CNV: Copy Number Variant
DNA: Deoxyribonucleic Acid
FFPE: Formalin-Fixed Paraffin-Embedded
FISH: Fluorescence *In Situ* Hybridization
IHC: Immunohistochemistry
INDEL: Insertion Deletion
IGV: Integrative Genomics Viewer
LDTs: Laboratory-Developed Tests
LOD: Limit of Detection
MSI: Microsatellite Instability
MSI-H: Microsatellite Instable High
MSI-S: Microsatellite Stable
NGS: Next Generation Sequencing
PCR: polymerase chain reaction
RNA: Ribonucleic Acid
SNV: Single Nucleotide Variant
SSV: Splice Site Variant
SV: Structural Variant
TMB: Tumor Mutation Burden
WES: whole exome sequencing

## References

[1] Siegel RL , Miller KD , Fuchs HE , Jemal A . Cancer Statistics, 2021. CA Cancer J Clin. 2021; 71:7–33. 10.3322/caac.21654. 33433946

[2] El-Deiry WS , Goldberg RM , Lenz HJ , Shields AF , Gibney GT , Tan AR , Brown J , Eisenberg B , Heath EI , Phuphanich S , Kim E , Brenner AJ , Marshall JL . The current state of molecular testing in the treatment of patients with solid tumors, 2019. CA Cancer J Clin. 2019; 69:305–43. 10.3322/caac.21560. 31116423 PMC6767457

[3] Malone ER , Oliva M , Sabatini PJB , Stockley TL , Siu LL . Molecular profiling for precision cancer therapies. Genome Med. 2020; 12:8. 10.1186/s13073-019-0703-1. 31937368 PMC6961404

[4] Wheler JJ , Janku F , Naing A , Li Y , Stephen B , Zinner R , Subbiah V , Fu S , Karp D , Falchook GS , Tsimberidou AM , Piha-Paul S , Anderson R , et al. Cancer Therapy Directed by Comprehensive Genomic Profiling: A Single Center Study. Cancer Res. 2016; 76:3690–701. 10.1158/0008-5472.CAN-15-3043. 27197177

[5] Agarwala V , Khozin S , Singal G , O'Connell C , Kuk D , Li G , Gossai A , Miller V , Abernethy AP . Real-World Evidence In Support Of Precision Medicine: Clinico-Genomic Cancer Data As A Case Study. Health Aff (Millwood). 2018; 37:765–72. 10.1377/hlthaff.2017.1579. 29733723

[6] Gutierrez ME , Choi K , Lanman RB , Licitra EJ , Skrzypczak SM , Pe Benito R , Wu T , Arunajadai S , Kaur S , Harper H , Pecora AL , Schultz EV , Goldberg SL . Genomic Profiling of Advanced Non-Small Cell Lung Cancer in Community Settings: Gaps and Opportunities. Clin Lung Cancer. 2017; 18:651–59. 10.1016/j.cllc.2017.04.004. 28479369

[7] Frampton GM , Fichtenholtz A , Otto GA , Wang K , Downing SR , He J , Schnall-Levin M , White J , Sanford EM , An P , Sun J , Juhn F , Brennan K , et al. Development and validation of a clinical cancer genomic profiling test based on massively parallel DNA sequencing. Nat Biotechnol. 2013; 31:1023–31. 10.1038/nbt.2696. 24142049 PMC5710001

[8] Garofalo A , Sholl L , Reardon B , Taylor-Weiner A , Amin-Mansour A , Miao D , Liu D , Oliver N , MacConaill L , Ducar M , Rojas-Rudilla V , Giannakis M , Ghazani A , et al. The impact of tumor profiling approaches and genomic data strategies for cancer precision medicine. Genome Med. 2016; 8:79. 10.1186/s13073-016-0333-9. 27460824 PMC4962446

[9] Halperin RF , Carpten JD , Manojlovic Z , Aldrich J , Keats J , Byron S , Liang WS , Russell M , Enriquez D , Claasen A , Cherni I , Awuah B , Oppong J , et al. A method to reduce ancestry related germline false positives in tumor only somatic variant calling. BMC Med Genomics. 2017; 10:61. 10.1186/s12920-017-0296-8. 29052513 PMC5649057

[10] Landrum MJ , Lee JM , Benson M , Brown GR , Chao C , Chitipiralla S , Gu B , Hart J , Hoffman D , Jang W , Karapetyan K , Katz K , Liu C , et al. ClinVar: improving access to variant interpretations and supporting evidence. Nucleic Acids Res. 2018; 46:D1062–67. 10.1093/nar/gkx1153. 29165669 PMC5753237

[11] Parikh K , Huether R , White K , Hoskinson D , Beaubier N , Dong H , Adjei AA , Mansfield AS . Tumor Mutational Burden From Tumor-Only Sequencing Compared With Germline Subtraction From Paired Tumor and Normal Specimens. JAMA Netw Open. 2020; 3:e200202. 10.1001/jamanetworkopen.2020.0202. 32108894 PMC7049088

[12] Beaubier N , Tell R , Lau D , Parsons JR , Bush S , Perera J , Sorrells S , Baker T , Chang A , Michuda J , Iguartua C , MacNeil S , Shah K , et al. Clinical validation of the tempus xT next-generation targeted oncology sequencing assay. Oncotarget. 2019; 10:2384–96. 10.18632/oncotarget.26797. 31040929 PMC6481324

[13] Freedman AN , Klabunde CN , Wiant K , Enewold L , Gray SW , Filipski KK , Keating NL , Leonard DGB , Lively T , McNeel TS , Minasian L , Potosky AL , Rivera DR , et al. Use of Next-Generation Sequencing Tests to Guide Cancer Treatment: Results from a Nationally Representative Survey of Oncologists in the United States. JCO Precision Oncology. 2018; 1–13. 35135159 10.1200/PO.18.00169 PMC9797241

[14] Gong J , Pan K , Fakih M , Pal S , Salgia R . Value-based genomics. Oncotarget. 2018; 9:15792–815. 10.18632/oncotarget.24353. 29644010 PMC5884665

[15] Avila M , Meric-Bernstam F . Next-generation sequencing for the general cancer patient. Clin Adv Hematol Oncol. 2019; 17:447–54. 31449513 PMC6739831

[16] Adashek JJ , Kato S , Parulkar R , Szeto CW , Sanborn JZ , Vaske CJ , Benz SC , Reddy SK , Kurzrock R . Transcriptomic silencing as a potential mechanism of treatment resistance. JCI Insight. 2020; 5:e134824. 10.1172/jci.insight.134824. 32493840 PMC7308055

[17] Thorvaldsdóttir H , Robinson JT , Mesirov JP . Integrative Genomics Viewer (IGV): high-performance genomics data visualization and exploration. Brief Bioinform. 2013; 14:178–92. 10.1093/bib/bbs017. 22517427 PMC3603213

[18] Bonneville R , Krook MA , Kautto EA , Miya J , Wing MR , Chen HZ , Reeser JW , Yu L , Roychowdhury S . Landscape of Microsatellite Instability Across 39 Cancer Types. JCO Precis Oncol. 2017; 2017:PO.17.00073. 10.1200/PO.17.00073. 29850653 PMC5972025

[19] Chalmers ZR , Connelly CF , Fabrizio D , Gay L , Ali SM , Ennis R , Schrock A , Campbell B , Shlien A , Chmielecki J , Huang F , He Y , Sun J , et al. Analysis of 100,000 human cancer genomes reveals the landscape of tumor mutational burden. Genome Med. 2017; 9:34. 10.1186/s13073-017-0424-2. 28420421 PMC5395719

[20] Tokunaga R , Xiu J , Johnston C , Goldberg RM , Philip PA , Seeber A , Naseem M , Lo JH , Arai H , Battaglin F , Puccini A , Berger MD , Soni S , et al. Molecular Profiling of Appendiceal Adenocarcinoma and Comparison with Right-sided and Left-sided Colorectal Cancer. Clin Cancer Res. 2019; 25:3096–103. 10.1158/1078-0432.CCR-18-3388. 30692096 PMC6886223

[21] Abdel-Wahab R , Yap TA , Madison R , Pant S , Cooke M , Wang K , Zhao H , Bekaii-Saab T , Karatas E , Kwong LN , Meric-Bernstam F , Borad M , Javle M . Genomic profiling reveals high frequency of DNA repair genetic aberrations in gallbladder cancer. Sci Rep. 2020; 10:22087. 10.1038/s41598-020-77939-6. 33328484 PMC7745036

[22] Matsubara T , Soh J , Morita M , Uwabo T , Tomida S , Fujiwara T , Kanazawa S , Toyooka S , Hirasawa A . DV200 Index for Assessing RNA Integrity in Next-Generation Sequencing. Biomed Res Int. 2020; 2020:9349132. 10.1155/2020/9349132. 32185225 PMC7063185

[23] Iranzo J , Martincorena I , Koonin EV . Cancer-mutation network and the number and specificity of driver mutations. Proc Natl Acad Sci U S A. 2018; 115:E6010–E6019. 10.1073/pnas.1803155115. 29895694 PMC6042135

[24] ICGC/TCGA Pan-Cancer Analysis of Whole Genomes Consortium. Pan-cancer analysis of whole genomes. Nature. 2020; 578:82–93. 10.1038/s41586-020-1969-6. 32025007 PMC7025898

[25] Martincorena I , Raine KM , Gerstung M , Dawson KJ , Haase K , Van Loo P , Davies H , Stratton MR , Campbell PJ . Universal Patterns of Selection in Cancer and Somatic Tissues. Cell. 2018; 173:1823. 10.1016/j.cell.2018.06.001. 29906452 PMC6005233

[26] Essakly A , Loeser H , Kraemer M , Alakus H , Chon SH , Zander T , Buettner R , Hillmer AM , Bruns CJ , Schroeder W , Gebauer F , Quaas A . PIK3CA and KRAS Amplification in Esophageal Adenocarcinoma and their Impact on the Inflammatory Tumor Microenvironment and Prognosis. Transl Oncol. 2020; 13:157–64. 10.1016/j.tranon.2019.10.013. 31865178 PMC6931191

[27] Khatami F , Tavangar SM . A Review of Driver Genetic Alterations in Thyroid Cancers. Iran J Pathol. 2018; 13:125–35. 30697281 PMC6339486

[28] Younis E . Oncogenesis of Thyroid Cancer. Asian Pac J Cancer Prev. 2017; 18:1191–99. 10.22034/APJCP.2017.18.5.1191. 28610401 PMC5555522

[29] Weinstein JN , Collisson EA , Mills GB , Shaw KR , Ozenberger BA , Ellrott K , Shmulevich I , Sander C , Stuart JM , and Cancer Genome Atlas Research Network. The Cancer Genome Atlas Pan-Cancer analysis project. Nat Genet. 2013; 45:1113–20. 10.1038/ng.2764. 24071849 PMC3919969

[30] Galipeau PC , Prevo LJ , Sanchez CA , Longton GM , Reid BJ . Clonal expansion and loss of heterozygosity at chromosomes 9p and 17p in premalignant esophageal (Barrett's) tissue. J Natl Cancer Inst. 1999; 91:2087–95. 10.1093/jnci/91.24.2087. 10601379 PMC1559996

[31] Hu N , Huang J , Emmert-Buck MR , Tang ZZ , Roth MJ , Wang C , Dawsey SM , Li G , Li WJ , Wang QH , Han XY , Ding T , Giffen C , et al. Frequent inactivation of the TP53 gene in esophageal squamous cell carcinoma from a high-risk population in China. Clin Cancer Res. 2001; 7:883–91. 11309337

[32] Shain AH , Salari K , Giacomini CP , Pollack JR . Integrative genomic and functional profiling of the pancreatic cancer genome. BMC Genomics. 2013; 14:624. 10.1186/1471-2164-14-624. 24041470 PMC3848637

[33] Cicenas J , Kvederaviciute K , Meskinyte I , Meskinyte-Kausiliene E , Skeberdyte A , Cicenas J . KRAS, TP53, CDKN2A, SMAD4, BRCA1, and BRCA2 Mutations in Pancreatic Cancer. Cancers (Basel). 2017; 9:42. 10.3390/cancers9050042. 28452926 PMC5447952

[34] Zeng H , Jorapur A , Shain AH , Lang UE , Torres R , Zhang Y , McNeal AS , Botton T , Lin J , Donne M , Bastian IN , Yu R , North JP , et al. Bi-allelic Loss of CDKN2A Initiates Melanoma Invasion via BRN2 Activation. Cancer Cell. 2018; 34:56–68.e9. 10.1016/j.ccell.2018.05.014. 29990501 PMC6084788

[35] Liu H , Zhang B , Sun Z . Spectrum of EGFR aberrations and potential clinical implications: insights from integrative pan-cancer analysis. Cancer Commun (Lond). 2020; 40:43–59. 10.1002/cac2.12005. 32067422 PMC7163653

[36] Kandoth C , Schultz N , Cherniack AD , Akbani R , Liu Y , Shen H , Robertson AG , Pashtan I , Shen R , Benz CC , Yau C , Laird PW , Ding L , et al, and Cancer Genome Atlas Research Network. Integrated genomic characterization of endometrial carcinoma. Nature. 2013; 497:67–73. 10.1038/nature12113. 23636398 PMC3704730

[37] Risinger JI , Hayes K , Maxwell GL , Carney ME , Dodge RK , Barrett JC , Berchuck A . PTEN mutation in endometrial cancers is associated with favorable clinical and pathologic characteristics. Clin Cancer Res. 1998; 4:3005–10. 9865913

[38] Chen T , Wang Z , Zhou W , Chong Z , Meric-Bernstam F , Mills GB , Chen K . Hotspot mutations delineating diverse mutational signatures and biological utilities across cancer types. BMC Genomics. 2016 (Suppl 2); 17:394. 10.1186/s12864-016-2727-x. 27356755 PMC4928158

[39] Tomczak K , Czerwińska P , Wiznerowicz M . The Cancer Genome Atlas (TCGA): an immeasurable source of knowledge. Contemp Oncol (Pozn). 2015; 19:A68–77. 10.5114/wo.2014.47136. 25691825 PMC4322527

[40] Hasselblatt M , Riesmeier B , Lechtape B , Brentrup A , Stummer W , Albert FK , Sepehrnia A , Ebel H , Gerss J , Paulus W . BRAF-KIAA1549 fusion transcripts are less frequent in pilocytic astrocytomas diagnosed in adults. Neuropathol Appl Neurobiol. 2011; 37:803–06. 10.1111/j.1365-2990.2011.01193.x. 21696415

[41] Banerjee A , Jakacki RI , Onar-Thomas A , Wu S , Nicolaides T , Young Poussaint T , Fangusaro J , Phillips J , Perry A , Turner D , Prados M , Packer RJ , Qaddoumi I , et al. A phase I trial of the MEK inhibitor selumetinib (AZD6244) in pediatric patients with recurrent or refractory low-grade glioma: a Pediatric Brain Tumor Consortium (PBTC) study. Neuro Oncol. 2017; 19:1135–44. 10.1093/neuonc/now282. 28339824 PMC5570236

[42] Burris HA , Saltz LB , Yu PP . Assessing the Value of Next-Generation Sequencing Tests in a Dynamic Environment. Am Soc Clin Oncol Educ Book. 2018; 38:139–46. 10.1200/EDBK_200825. 30231307

[43] Qin D . Next-generation sequencing and its clinical application. Cancer Biol Med. 2019; 16:4–10. 31119042 10.20892/j.issn.2095-3941.2018.0055 PMC6528456

[44] Caris Life Sciences https://www.carislifesciences.com/molecular-profiling-technology/. Accessed February 26, 2021.

[45] Tempus https://www.tempus.com/genomic-profiling/. February 26, 2021.

[46] Chan TA , Yarchoan M , Jaffee E , Swanton C , Quezada SA , Stenzinger A , Peters S . Development of tumor mutation burden as an immunotherapy biomarker: utility for the oncology clinic. Ann Oncol. 2019; 30:44–56. 10.1093/annonc/mdy495. 30395155 PMC6336005

[47] Murciano-Goroff YR , Drilon A , Stadler ZK . The NCI-MATCH: A National, Collaborative Precision Oncology Trial for Diverse Tumor Histologies. Cancer Cell. 2021; 39:22–24. 10.1016/j.ccell.2020.12.021. 33434511 PMC10640715

