# Supplementary material for: Analytic validation and clinical utilization of the comprehensive genomic profiling test, GEM ExTra®
Source: Oncotarget. 2021 Apr 13;12(8):726–39. doi: 10.18632/oncotarget.27945 (PMC8057276; doi:10.18632/oncotarget.27945)
Supplement: Supplementary file 1 [file oncotarget-12-726-s001.pdf]

Analytic validation and clinical utilization of the comprehensive genomic profiling test, GEM ExTra®

SUPPLEMENTARY MATERIALS

Supplementary Table 1: Hotspot mutations. See Supplementary Table 1

Supplementary Table 2: RNA standard reference comparison and patient tumor sample orthogonal testing

Sample ID	Orthogonal Method	Orthogonal Results	GEM ExTra Results
C007-0114	PCR	MSI stable	MSI stable
C020-0092	PCR	MSI stable	MSI stable
C020-0098	PCR	MSI stable	MSI stable
C020-0100	PCR	MSI stable	MSI stable
C020-0101	PCR	MSI stable	MSI stable
C020-0103	PCR	MSI stable	MSI stable
C020-0105	PCR	MSI stable	MSI stable
C020-0108	PCR	MSI stable	MSI stable
C020-0128	PCR	MSI stable	MSI stable
C020-0131	PCR	MSI stable	MSI stable
C020-0133	PCR	MSI stable	MSI stable
C020-0135	PCR	MSI stable	MSI stable
C020-0139	PCR	MSI stable	MSI stable
C020-0150	PCR	MSI stable	MSI stable
C020-0151	PCR	MSI stable	MSI stable
C020-0162	IHC	MLH1, PMS 2 lost	MSI high
C020-0174	IHC	MSI high	MSI high
C020-0175	IHC	MSI high	MSI high
C020-0177	IHC	MSI high	MSI high
C020-0183	PCR	MSI high	MSI high
C020-0184	IHC	MSI stable	MSI stable
C020-0185	PCR	MSI high	MSI high
C020-0186	IHC	MSI stable	MSI stable
C020-0187	PCR	MSI high	MSI high
C023-0119	PCR	MSI stable	MSI stable
C028-0045	PCR	MSI high	MSI high
C030-0001	PCR	MSI stable	MSI stable
C030-0220	PCR	MSI high	MSI high
C030-0288	PCR	MSI high	MSI high

Supplementary Table 3: Summary of clinical requisitions for GEM ExTra assay 2018–2019

	Patients Tested	Clinical Reports	DNA + RNA	DNA only	Single Biopsy Patients	>1 Biopsies Patients
**2018**	351	369	315	54	334	16
**2019**	1098	1140	946	194	1049	49
**Total**	1435	1509	1261	248	1383	65
