# Supplementary material for: Analytic validation and clinical utilization of the comprehensive genomic profiling test, GEM ExTra®
Source: Oncotarget. 2021 Apr 13;12(8):726–39. doi: 10.18632/oncotarget.27945 (PMC8057276; doi:10.18632/oncotarget.27945)
Supplement: Supplementary file 2 [file oncotarget-12-726-s002.docx]

**Supplementary Table 1: Hotspot mutations**

| **COSMIC Gene Symbol** | **Mutations** |
| --- | --- |
| ABCG2_ENST000002376 12 | Q141K |
| ABL1 | D276G,E255K,E255V,E355G,F317L,F359C,F359I,F359V,G250E,H396R,K247R,L248V,L38 7M,M244V,M351T,Q252H,T315I,Y253F,Y253H |
| ACVR1 | G328E,G328V,R258G |
| AKT1 | E17K,E40K,L52R,Q79K,W80R |
| AKT2 | E17K,V140A |
| AKT3 | E17K |
| ALK | C1156Y,D1091N,F1174C,F1174I,F1174L,F1174S,F1174V,F1245C,F1245I,F1245L,F1245V,G1202R,G1269A,I1171N,I1171T,L1196M,R1275*,R1275L,R1275Q,T1151M,Y1278S |
| APC | C1578fs*71,E1284*,E1286*,E1306*,E1309*,E1309K,E1309fs*4,E1309fs*6,E1317*,E1317Q,E1317fs*4,E1379*,E1464*,E1464fs*8,E1494fs*13,E1494fs*19,F1491fs*16,F1491fs*17,G1339A,I1307K,I1307fs*1,I1307fs*14,I1307fs*6,I1307fs*8,I1557fs*1,I1557fs*2,K1555fs*3,L1488*,L1488fs*18,L1488fs*19,L1488fs*23,L1488fs*24,L1488fs*25,L1488fs*26,N1455fs*18,N1455fs*2,P1319fs*2,P1361L,P1361S,P1361fs*13,P1372fs*2,P1373fs*42,P1439fs*34,P1440fs*33,P1453fs*20,Q1338*,Q1367*,Q1378*,Q1429*,R1114*,R1399fs*16,R1399fs*9,R1450*,R213*,R216*,R2166*,R2166Q,R876*,S1234fs*31,S1341R,S1400*,S1400L,S1400fs*1,S1400fs*15,S1407fs*1,S1407fs*8,S1411fs*4,S1415fs*4,S1415fs*8,S1421fs*1,S1421fs*52,S1465fs*3,S1465fs*9,T1438fs*35,T1445fs*27,T1445fs*28,T1487fs*17,T1487fs*27,T1493T,T1493fs*14,T1537K,T1556fs*3,T1556fs*9,V1414I, V1414fs*1,V1414fs*5,V1414fs*9 |
| AR | F877L,H875Y,L702H,T878A,T878S,W742C |
| ARAF | S214F |
| ARID1A | D1850fs*33,D1850fs*4,G2087E,G2087R,Q588* |
| ARID2 | A1773V,R285Q,R285W,R314C,R314S,S297F |
| ASXL1 | E1102D,E635fs*15,E635fs*68,G645fs*58,G646fs*12,G646fs*57,Y591*,Y591fs*1 |
| ASXL2_ENST0000043550 4 | R591C |
| ATM | D1853V,E2164K,E2164Q,L2427P,L2890V,N2875T,P2699S,R2691C,R2832C,R3008H,R33 7C,T2666A,V1941L |
| ATM_ENST00000278616 | D1853N,R3008C |
| ATR | I774fs*3,I774fs*5 |
| ATRX | R1512H,R1514* |
| B2M | M1I,M1L,M1R,M1T |
| B2M_ENST00000544417 | M1V |
| BCL2L12 | R18W |
| BCL6 | N14255 |
| BCOR | N1425S |
| BRAF | D594A,D594G,D594H,D594N,D594V,E586K,F595L,F595S,G464E,G464R,G464V,G466A, G466E,G466R,G466V,G469A,G469E,G469R,G469S,G469V,G596D,G596R,I592M,K601E, K601I,K601N,K601T,K601del,L597Q,L597R,L597S,L597V,N581S,N581T,T599I,V471F,V6 00A,V600D,V600E,V600G,V600K,V600L,V600M,V600R,Y472C |
| BRCA2 | I605fs*9,K944*,S455L,S455S |
| BTK | C481R,C481S |
| C15orf23_ENST0000041 6151 | S24F |
| CARD11 | R170C,R170H |
| CBL | C384R,C384Y,C404Y,L380P,R420Q,Y371C,Y371D,Y371H |
| CCND1 | P287S,T286I |
| CCND3 | I290R,P284L,T283P |
| CDH1 | T263fs*3 |
| CDK4 | R24C,R24H,R24L,R24S |
| CDKN2A | A57P,A57V,A60V,A68A,A68T,A68V,D74A,D74N,D74Y,D84D,D84V,E88K,G101W,G67S,H83N,H83P,L97P,L97R,M53I,N71I,P114H,P114L,P114S,P48L,P48R,P81S,P81fs*38,Q50*, Q50R,R112G,R24P,R58*,R80Q,S43I,W110* |
| CDKN2A_ENST00000361 570 | A138A,A138G |
| CDKN2A_ENST00000446 177 | A57T,D108N,D108Y,D84G,D84N,D84Y,E61*,E88*,P81L,R80* |
| CDKN2A_ENST00000498 124 | A57A,D108G,D108H,E69*,H83Y,L78fs*41,P114P,Q50H,W110* |
| CEBPA | E309_T310insE,E309_T310insK,E316_L317insQ,E316_L317insR,H24fs*84,K304_Q305insK,K304_Q305insL,K313>EK,K313_V314>insE,K313_V314insK,K313_V314insRNVETQQK,K313del,P23fs*137,P23fs*81,Q312_K313insE,Q312_K313insQ,Q83fs*26,Q83fs*77 |
| CHEK2 | K373E |
| CIC | R215Q,R215W |
| CREBBP | R1446C,R1446G,R1446H,R1446L,S1680delS |
| CRLF2 | F232C |
| CRNKL1 | S128F |
| CSF1R | L301S,Y969*,Y969C,Y969F,Y969H |
| CSF3R_ENST0000037310 4 | T618I |
| CTCF | R377C,R377H |
| CTNNB1 | A13T,A21T,A43T,A43V,D32A,D32E,D32G,D32H,D32N,D32V,D32Y,E53K,G34E,G34R,G34V,G48D,H36P,H36R,H36Y,I35I,I35S,I35T,I35_G38delIHSG,K335I,K335T,K49*,K49R,N387K,P44A,P44L,P44S,S33A,S33C,S33F,S33L,S33N,S33P,S33S,S33Y,S37A,S37C,S37F,S37P,S37Y,S45A,S45C,S45F,S45P,S45T,S45Y,S45_S47>C,S45del,T40I,T41A,T41I,T41N,T41P,T41S,T42I,T42R,T42T,T42_A43insSS,T42fs*7,V22A,V22_G38del,W383R,WQQQSYLD25? |
| CXCR4 | G336fs*12,R338*,S342fs*15,S342fs*6 |
| DDR2 | S768R |
| DICER1 | E1813A,E1813D,E1813G,E1813K,E1813Q |
| DIS3 | D488N,R382Q |
| DNMT1 | E432K |
| DNMT3A | F731L,F731del,G543A,G543C,G543V,R635Q,R635W,R882H,R882L,S714C,W893S |
| DNMT3A_ENST0000038 0746 | R693C,R693P,R693S |
| DOT1L_ENST000003986 65 | G1386S |
| EGFR | A289D,A289T,A289V,A750P,A839T,A859T,D761N,D761Y,D770_N771insG,D770_N771insGF,D770_N771insSVD,E709A,E709G,E709K,E709V,E746K,E746_A750delELREA,G719A,G719C,G719D,G719S,G724S,G735S,G796S,G863D,G874S,H773R,H773_V774insH,H773_V774insNPH,H773_V774insPH,H835L,L747P,L747S,L747_E749delLRE,L747_S752delLREATS,L833F,L833V,L838P,L858L,L858M,L858R,L861Q,L861R,P596L,P733L,P753L,P753S,P848L,R108K,R222C,R252H,R252P,R776C,R776H,S492R,S768I,T790M,T847I,T854A,T854S,V689M,V742A,V769L,V774_C775insHV,V843I,W731* |
| EGFR_ENST0000034291 6 | R252C |
| EGFR_ENST0000034457 6 | T263P |
| EGFR_ENST0000044259 1 | G598A,G598V |
| EP300 | C1164Y,D1399N,D1399Y |
| EPHA5 | G582E |
| ERBB2 | A293T,A775_G776insYVMA,E770_A771insAYVM,E930D,G660D,G776>VC,G776S,G776V,I767M,L755M,L869R,P780_Y781insGSP,R896C,T733I,T862A,V659E,V697L,V777L,V77 7M |
| ERBB2_ENST000005417 74 | D754H,D754Y,L740P,L740S,R663Q,S295F,S295Y,V762L,V827I |
| ERBB3 | A232V,D297Y,E928G,G284R,P262H,Q809R,S846I,V104L |
| ERBB3_ENST000002671 01 | V104M |
| ERBB3_ENST000004117 31 | M91I,V104L |
| ERBB4 | E452K,E542K,R393W,R544W,R711C |
| ERBB4_ENST000004025 97 | E862K |
| ERCC2_ENST0000039194 5 | D312N,K751Q |
| ESR1 | D538G,L536H,L536P,L536R,S463P,Y537C,Y537N,Y537S |
| ESR1_ENST00000544394 | E207Q |
| ETV1 | R187C |
| ETV6 | R369W |
| EZH2 | A682G,R690C,R690H,Y646C,Y646F |
| EZH2_ENST00000350995 | Y602H,Y602N,Y602S |
| FBXW7 | G423R,R222*,R224*,R224Q,R278*,R465H,R465fs*7,R479G,R479L,R505?,R505P,R505S,R689Q,S282*,S596F |
| FBXW7_ENST000002817 08 | G423V,R465L,R465S,R465fs*7,R479*,R479P,R479Q,R479fs*6,R505C,R505H,R689W,S5 82L |
| FBXW7_ENST000003939 56 | R289C |
| FBXW7_NM_018315_2 | R425G,R425L,R578Q |
| FCGR2A | H166R |
| FCGR3A | F212V |
| FGFR1 | N546K,S125L |
| FGFR1_ENST0000044771 2 | N546K |
| FGFR2 | D101Y,K659E,K659N,R203C |
| FGFR2_ENST0000035193 6 | N547K |
| FGFR2_ENST0000035755 5 | C293R,N460K,Y286C |
| FGFR2_ENST0000036905 6 | N550H,N550S,W290C |
| FGFR2_ENST0000045741 6 | P253R,S252L,S252W |
| FGFR3 | A391E,A391V,G370C,G380R,G697C,K650M,K650Q,K650T,S249C,Y373C |
| FGFR3_ENST0000034010 7 | K652E,R248C,S373C |
| FGFR4_ENST0000029240 8 | N535K,V550L,V550M |
| FLT3 | D835A,D835E,D835F,D835H,D835N,D835V,D835Y,D835delD,D839G,F691L,I836M,I836 delI,N676K,N841K,V592A,Y572C,Y842C |
| FOXL2 | C134W |
| FUBP1_ENST000003707 67 | R430C |
| GATA1 | M1I,M1V,M1fs,S30fs*9,V74I |
| GATA2 | G320D,L321F,L321H,L321P,L321R,L321V,L359V,R362Q,T354M |
| GNA11 | Q209L,Q209P,Q209R,R183C |
| GNAQ | Q209H,Q209L,Q209P,Q209R,R183Q |
| GNAS | Q227H,Q227K,Q227L,Q227R,R201C,R201H,R201S |
| GNAS_ENST0000037110 0 | R844L |
| GRIN2A | R1067W |
| GSTP1 | I105V |
| H3F3A | K28M |
| HIST1H3B | E74K,E74Q |
| HNF1A | G292fs*25,P291fs*51,W206C,W206L |
| HRAS | E62G,G12A,G12R,G12V,G13R,G13S,G13V,Q61H,Q61P,Q61R |
| HRAS_ENST0000039759 4 | G12C,G12D,G12S,G13C,G13D,Q61K,Q61L |
| IDH1 | G70D,G70G,P33S,R132C,R132G,R132H,R132L,R132S,V178I,V71I |
| IDH2 | R140G,R140L,R140Q,R140W,R172G,R172K,R172M,R172S,R172T,R172W |
| IL7R | K395R,K395T,S185C |
| IRS2 | G1057D |
| JAK1 | R873H,V658F |
| JAK2 | E543_D544del,F537_K539>L,H538_K539>L,H538_K539>QL,I540_E543>MK,K539L,N54 2_E543del,R541_E543>K,R564L,R683G,R683S,R683T,T875N,V617F,V617I |
| JAK3 | A572T,A572V,A573V,R657Q |
| JAK3_ENST00000458235 | V722I |
| KDR | A1065T,R961W,S1100F |
| KEAP1 | R470C,R470H |
| KIT | A829P,D419G,D419N,D419_R420del,D419del,D52D,D52N,D572G,D572N,D579del,D816A,D816F,D816G,D816H,D816V,D816Y,D820E,D820G,D820H,D820V,D820Y,E554_K558del,E561K,E839K,K509I,K550_K558del,K558>NP,K558E,K558K,K558R,K558_E562del,K558_V559del,K558_V560>I,K558_V560>N,K558_V560del,K642E,K642Q,K818R,L576F,L576P,L576del,L862L,M541L,N655K,N822K,N822Y,P551_E554del,P551_M552>L,P551_V555del,Q556_V560>H,Q556_V560del,R888W,S715del,T670E,T670I,V555_I571del,V559A,V559D,V559G,V559I,V559_E561del,V559_G565del,V559_V560del,V559del,V560D,V560E,V560G,V560_L576del,V654A,V825A,V825I,W557C,W557G,W557R,W557S,W557_E561del,W557_K558>CP,W557_K558del,W557_V559>C,W557_V559>F,W557_V559del,W557_V560>C,W557del,Y503_F504insAY,Y553N,Y553_K558>,Y570_L576delYIDPT QL,Y823C,Y823D |
| KMT2C | V656I |
| KRAS | A146P,A146T,A146V,A59G,A59T,G10_A11insG,G12C,G12E,G12F,G12G,G12I,G12L,G12V,G12W,G12Y,G12_G13insG,G138G,G13D,G13E,G13F,G13G,G13N,G13R,G13S,G13V,G 13_V14insG,K117E,K117N,K117R,L19F,Q61E,Q61H,Q61K,T58I,V14G |
| KRAS_ENST00000256078 | A59E,G12A,G12C,G12D,G12R,G12S,G12V,G13A,G13C,G13D,G13V,K117N,L19F,Q22K,Q 61H,Q61K,Q61L,Q61P,Q61R,V14I |
| MAP2K1 | C121S,D67N,E203K,F53L,F53V,F53Y,G128D,G128V,I111S,K57E,K57N,K57T,P124L,P124 Q,P124S,Q56P |
| MAP2K2 | C125S,L46F,P298L |
| MAP2K2_ENST00000262 948 | Q60P |
| MAP2K4 | R134Q,R134W |
| MAP3K1_ENST00000399 503 | S1330L |
| MAPK1 | E322K |
| MED12 | G44S,G44V,G44_N46>D,G44_P49del,G44_Q48del,L1224F,L36P,L36_K42del,L36_Q43d el,Q43P,Q43_G44>R,Q43_G44del,Q43_N46>H |
| MED12_ENST000003336 46 | G44C |
| MED12_ENST000003740 80 | G44A,G44D,G44R,L36R |
| MEF2B | D83V |
| MET | D1028H,D1246N,F1218I,H1112R,H1112Y,M1268T,R988C,T1010I,V1110I,Y1248C,Y1248 H,Y1253D |
| MLH1 | V384D |
| MLL3_ENST0000035519 3 | K2797fs*26 |
| MPL | S505N,W515A,W515K,W515R,W515S |
| MPL_ENST00000413998 | W515L |
| MSH6 | F1088fs*2,F1088fs*3,F1088fs*5,T1219I |
| MTHFR_ENST000003765 83 | A263V |
| MTOR | C1483R,C1483Y,E1799K,E2419K,F1888I,I2500F,I2500M,L1433S,L1460P,L2209V,L2220F,R2505P,S2215F,S2215Y,T1977K |
| MYC | T58A,T58N |
| MYC_ENST00000377970 | A59V,T73I,T73P |
| MYCN | P44L,P44S,T58M |
| MYD88 | L265P,M232T,S243N |
| MYD88_ENST000004170 37 | S219C |
| NF1 | L844F,R1241* |
| NF2 | S288*,Y153fs*1 |
| NFE2L2 | D29G,D29H,D29N,D29Y,E79G,E79K,E79Q,E79V,E82D,E82G,E82Q,E82V,G31A,G31E,G31R,G81C,G81D,G81R,G81S,G81V,G81_F83delGEF,L30F,L30R,R34*,R34G,R34L,R34P,R34Q,T80A,T80I,T80K,T80P,T80R |
| NOTCH1 | C478F,D1517N,E450K,F1592S,G310R,G484V,L1593P,L1596H,L1600Q,L1678Q,L1678fs* 4,P1770S,P2514fs*4,P391S,R1598P,R2327W,R353C,R365C,S2467fs*10 |
| NOTCH1_ENST00000277 541 | A1944T,L1678P |
| NPM1 | W288fs*12,W288fs*>9,W290fs*10 |
| NQO1 | P187S |
| NRAS | A18T,G12A,G12C,G12D,G12N,G12R,G12S,G12V,G13A,G13C,G13D,G13G,G13N,G13R,G 13S,G13V,G60E,G60V,Q61E,Q61H,Q61K,Q61L,Q61P,Q61Q,Q61R |
| NT5C2_ENST000004234 68 | R338Q |
| NTRK1 | T264M |
| PAK7 | E144K |
| PAX5 | P80R |
| PDGFRA | D1071N,D842V,D842Y,D842_M844delDIM,D846Y,I843_D846delIMHD,N659K,N659Y,N 848K,S566_E571>K,S566_E571>R,T674I,V561D,V824V |
| PIK3C2G | S670P |
| PIK3CA | A1046E,A1046T,A1046V,C420R,C901F,D1045N,D549N,E418K,E453Q,E542A,E542G,E542Q,E542V,E545A,E545D,E545V,E547K,E726A,G1007D,G1007R,G1049A,G1049D,G1049G,G106R,G106V,G106_R108delGNR,H1047Q,H1047R,H1047Y,H701P,K111E,K111N,K111_I112>N,K567R,M1043I,N1044D,N1044K,N1044S,N1044Y,N1068fs*4,N1068fs*>2,N345H,N345S,N345T,N345Y,P471L,P539S,Q546E,Q546K,Q546L,Q546R,R108H,R38C,R38S,R93Q,S553fs*7,T1025?,T1025I,T1025N,T1025S,T1025T,V344A,V344M,Y1021C,Y1021 N |
| PIK3CA_ENST000002639 67 | A1066V,C378F,C378R,C378Y,E39K,E453K,E542K,E545G,E545K,E545Q,E726K,E81K,G1007V,G1049R,G1049S,G118D,H1047L,H1047Q,K111N,K111_I112delKI,K111delK,M1043L,M1043T,M1043V,N1044K,N345I,N345K,P539R,Q546H,Q546P,R38H,R88Q,R93W,T10 25A,T1052K,V344G,Y1021H |
| PIK3R1 | D560G,D560H,D560Y,D578fs*23,H450_E451del,K567E,K567_L570delKPDL,R162*,R30 1*,R348*,R358*,R386*,R461*,T576delT |
| PIK3R1_ENST000003206 94 | G76R |
| PIK3R1_ENST000003364 83 | N294D |
| PLCG2 | S707F |
| POLE | A456P,G364R,L424V,P286R,P441L,R114*,R1508C,R1519C,S297F,S459F,V411L |
| PPP2R1A | P179L,P179R,R182W,R183Q,R183W,R258H,S256F,S256Y,W257C,W257G |
| PPP6C_ENST0000045140 2 | R301C |
| PREX2_ENST0000035467 7 | G233C |
| PTCH1 | P1315L,Q853* |
| PTEN | A121P,A126D,A126P,A126S,A126T,A126V,A151T,A328fs*15,C105F,C105G,C105S,C105W,C105Y,C136F,C136R,C136Y,C136fs*44,C250fs*2,C71Y,D107Y,D92G,E242*,E242fs*15,F347fs*13,G127E,G129*,G129E,G129R,G129V,G165E,G165R,G165fs*9,H123Y,H61R,H93D,H93Q,H93R,H93Y,I101T,I67K,K128N,K128Q,K164fs*16,K164fs*3,K267fs*31,K267fs*9,K60fs*39,K66E,K66N,K6fs*4,L112P,L112V,L42R,N184fs*6,N323fs*2,P246L,P248?,P248fs*5,P38L,P38S,Q110*,Q149*,Q17*,Q171*,Q171E,Q171R,R130*,R130G,R130L,R130P,R130Q,R130_T131>P,R130fs*4,R159K,R159S,R173C,R173H,R233*,R234W,R335*,S170I,S170N,S170fs*13,T319del,T319fs*1,T319fs*24,T319fs*6,T321fs*23,V133I,V290fs*1,V290fs*8,Y155C,Y155H,Y68C,Y68H,Y68N,Y76delY,Y76fs*1,Y88C |
| PTPN11 | A72D,A72G,A72S,A72T,A72V,D61G,D61H,D61N,D61V,D61Y,E69K,E76A,E76G,E76K,E76Q,E76V,G503A,G503E,G503R,G503V,G60A,G60R,G60V,Q510H,Q510K,Q510L,S502A,S5 02L,S502P,T73I |
| PTPRD_ENST000003468 16 | S431L |
| PTPRD_ENST000003811 96 | P666S |
| Q7Z2S2_HUMAN | K656E |
| RAC1 | P29L |
| RAC1_ENST0000035614 2 | P29S |
| RAF1 | L613V,S259F |
| RAF1_ENST00000442415 | S257L |
| RB1 | C706F,E748*,L199*,R455*,R556* |
| RB1_ENST00000267163 | E137*,R320*,R358*,R552*,R579* |
| RET | A883F,C618Y,C630R,C634R,C634S,C634W,C634Y,D898_E901del,E632_L633del,E632_T 636>SS,E768D,M918T |
| RHEB | Y35C,Y35N |
| RHOA | E40Q,R5Q,R5W,Y42C,Y42S |
| RICTOR | S1101L |
| RIT1 | M90I |
| RQCD1 | P131L,S87C,S87P |
| RUNX1 | D198G,D198N,D198V,L56S,R107C,R107H,R162G,R162K,R162S,R201*,R201G,R201Q,R2 04*,R204Q |
| RUNX1T1 | D198N |
| RXRA | S427F,S427Y |
| SDHA | A466T,R465Q |
| SETBP1 | G870S |
| SF3B1 | E622D,G742D,H662D,H662Q,K666E,K666M,K666N,K666Q,K666R,K666T,K700E,R625C, R625G,R625H,R625L,R957Q |
| SH2B3 | E208Q |
| SMAD4 | A118V,D351G,D351H,D351N,D351Y,D537E,D537G,D537H,D537V,D537Y,E330A,G386D,G386R,G386S,G386V,P356L,P356R,P356S,R361C,R361G,R361H,R361S |
| SMARCA4 | G1232C,L1163P,T910M |
| SMARCA4_ENST0000035 8026 | G1232S |
| SMARCB1 | A382fs*5,P383fs*4,R377C,R377H,R377L |
| SMO | A459V,I408V,L412F,S533N,T241M,T640A,V321M,V404M,W535L |
| SOS1 | N233Y |
| SPOP | F102C,F102S,F102V,F133C,F133I,F133L,F133S,F133V,W131C,W131G,W131S,Y87C,Y87 N,Y87S |
| SRC | Q531* |
| SRSF2 | P107H,P95?,P95A,P95H,P95L,P95R,P95T,P95_R102del |
| STAG2 | R370Q |
| STAG2_ENST000002180 89 | R370W |
| STAT3 | D661V,D661Y,H410R,S614R,Y640F |
| STK11 | D194N,D194V,D194Y,F354L,K84*,P221L,P281L,Q170*,Q37*,S216F,W332* |
| STK11_ENST0000032687 3 | G196V,P281fs*6,R304W |
| STK19_ENST0000037533 3 | D89N |
| TET2 | A1355V,C1221Y,C1271S,C1271Y,C1289F,C1289Y,C1378Y,C25R,C262fs*31,F1287S,F868L,G1288D,H1380H,H1380Y,H1881R,H1904R,I1873T,L1322Q,P1419R,R1214W,R1261C, R1261H,T1884A,V1718L |
| TET2_ENST00000305737 | Q764fs*5 |
| TNFAIP3 | L324fs*7 |
| TP53 | A159A,A159D,A159S,A159T,A159fs*11,A161A,A161V,A161fs*9,A276S,A276T,A307fs*38,C135S,C141F,C141Y,C176S,C176fs*5,C176fs*71,C182S,C182Y,C229Y,C238*,C238S,C238W,C242*,C242C,C242S,C275C,C275F,C275S,C275fs*31,C277*,C277C,C277G,C277W,C277fs*68,D184H,D184N,D184Y,D184fs*24,D186G,D186H,D186N,D186fs*61,D208E,D208G,D208N,D208Y,D228D,D228E,D228G,D228H,D228N,D228Y,D228fs*1,D259E,D259G,D259H,D259N,D259V,D259fs*86,D281H,E171G,E171K,E171Q,E171fs*3,E180*,E198K,E198fs*49,E221*,E221K,E221fs*26,E221fs*4,E224D,E224K,E258Q,E271G,E285E,E285G,E285Q,E285V,E286D,E286V,E287D,E287E,E287K,E294E,E294K,E294fs*12,E298*,E298fs*46,E336*,E336fs*10,E339*,E343*,E343fs*2,E346*,E349*,E349fs*21,F109V,F113C,F113V,F212fs*3,F270L,F270Y,G154D,G154G,G154I,G154S,G154fs*16,G187V,G199E,G199R,G226D,G226S,G226V,G244A,G244G,G245C,G245G,G245N,G245V,G245fs*2,G262D,G262fs*83,G266A,G266E,G266R,G266fs*79,G279G,G279R,G279V,G334V,G334W,H168H,H168P,H168Y,H178N,H178Q,H178Y,H179Q,H179Y,H193fs*16,I162M,I162V,I195S,I195fs*52,I232N,I232V,I251I,I251L,I251T,I251fs*94,I254F,I254N,I254T,I254V,I254fs*10,I255V,I255fs*9,I255fs*90,K164K,K164M,K164N,K291*,K291E,K291K,K291N,K291R,K291T,K291fs*48,K292R,K292T,K320K,K320N,L111P,L111Q,L145L,L145P,L194H,L194L,L194R,L194V,L252F,L252_I254delLTI,L257L,L257R,L257V,L257fs*6,L265L,L265M,L265R,L265delL,L265fs*80,M160I,M160K,M160L,M160V,M169I,M169T,M169V,M237L,M237R,M237fs*10,M243I,M243L,M243T,M243V,M246I,M246L,M246fs*1,N200fs*47,N235I,N235S,N235T,N235delN,N239K,N239S,N239Y,N239_C242delNSSC,N239_S240delNS,N239_S240insN,N239fs*1,N239fs*25,N247D,N247N,N247S,N247T,N247Y,N288S,N288Y,N288fs*13,N288fs*57,P151L,P151P,P151fs*30,P152P,P152Q,P152R,P152fs*29,P153L,P153P,P153S,P153T,P153fs*28,P177H,P177P,P190F,P191L,P191S,P191fs*56,P250F,P250P,P250S,P278R,P278S,P278T,P300L,P300S,P301S,P47S,Q136H,Q136P,Q136Q,Q136fs*34,Q144H,Q144L,Q192H,Q192R,R156G,R156L,R156S,R156fs*14,R156fs*25,R158C,R158R,R158S,R158_A159delRA,R158_A159insX,R158fs*11,R158fs*12,R174G,R174K,R174fs*73,R175P,R175S,R181C,R181H,R181L,R196*,R196L,R196Q,R196R,R196fs*51,R202C,R202H,R202L,R202S,R209*,R209I,R209K,R209T,R209fs*38,R213L,R213P,R213Q,R213fs*34,R248Q,R248R,R248fs*97,R249R,R249S,R249T,R249fs*96,R267G,R267P,R267R,R273C,R273P,R280K,R280R,R280S,R280fs*65,R282L,R282Q,R282R,R282W,R282fs*63,R283C,R283L,R283fs*62,R290L,R290fs*55,R337C,R337H,R337L,R337S,R342*,R342P,R342fs*3,S149F,S149P,S149S,S149fs*32,S166L,S166P,S215C,S215R,S215T,S227F,S240C,S240I,S240R,S241A,S241F,S241P,S241delS,S241fs*6,S303N,S303T,S303fs*42,T125P,T125R,T155A,T155T,T155_R156delTR,T170M,T170T,T211A,T211I,T211N,T211T,T211fs*36,T211fs*4,T230I,T230fs*17,T253A,T253I,T253N,T253S,T253T,T284A,T284P,T284T,V122fs*26,V143M,V147A,V147D,V147I,V147V,V157A,V157D,V157G,V157I,V157L,V157V,V157fs*13,V157fs*23,V172A,V172D,V172F,V172G,V172I,V172fs*2,V173V,V173fs*1,V173fs*7,V197A,V197E,V197G,V197L,V203E,V203M,V216A,V216E,V216G,V217A,V217E,V218A,V218E,V218G,V218M,V272A,V272fs*73,V274A,V274G,V274I,V73fs*50,V73fs*76,Y126N,Y163*,Y163H,Y163S,Y163Y,Y205*,Y220*,Y220S,Y234N,Y234delY,Y236*,Y 236S,Y236delY |
| TP53BP1_ENST00000382 044 | P1775S |
| TP53_ENST00000269305 | A276G,C135F,C141R,C176R,C176W,C182*,C229*,C238F,C242F,C242R,C242S,C275*,C275W,C275fs*70,C277F,D281D,D281E,D281G,E224*,E258A,E258D,E285*,E286*,E286K,E287D,E294*,E294fs*51,F109C,F270C,F270I,F270S,G262V,G266*,G266V,G279E,H168R,H178fs*69,H179Q,H193R,I251N,I254S,I255delI,K132M,K132N,K132Q,K132R,K292*,K320*,L194P,L252delL,L252fs*93,L257P,L265P,M237I,M246I,M246R,M246T,N247I,P151P,P177L,P177R,P177S,P278A,P278F,P278L,P278fs*67,Q136E,Q144P,R158G,R181P,R196*,R196P,R213*,R248L,R248P,R249M,R267W,R273G,R273L,R280*,R280I,R280T,R282W,R306*,S166*,S241T,S241Y,T125K,T211P,T230P,V147fs*23,V173L,V272M,V274F,Y22 0fs*27,Y236H |
| TP53_ENST00000413465 | A161D,A161S,C176*,C238G,C238R,C238S,C242W,D184fs*62,E171*,E180D,E224D,E224E,E258*,E258D,E258K,E258V,G187fs*60,G244C,G244fs*3,G245D,G245R,H178fs*3,H179D,H179Y,H193Y,I162N,I232F,I232T,I232fs*15,I251F,K164E,L257Q,M237V,M246V,N239T,P151R,P151S,P151T,P152fs*14,P190R,P190T,P190fs*57,Q136*,Q192*,R156H,R156P,R158L,R175G,R175H,R248W,R249K,S149fs*21,S215I,S215R,T155I,T155N,V173G,V173fs*1,Y205H,Y234*,Y234H |
| TP53_ENST00000414315 | C3G,C3R,C3Y,C9G,C9W,H47L,H61D,H61N,I63F,I63T,V41E,V41M,V84M,Y31D,Y73D,Y73 N,Y73S |
| TP53_ENST00000420246 | A159V,A161T,A276V,C238Y,C242G,C242fs*5,C275R,C275Y,D259Y,D281N,D281V,E180K,E271K,E271Q,E271V,E285K,E286A,E286G,E286Q,E287*,E294fs*>48,F109S,F270V,G154fs*16,G187D,G187R,G187S,G199*,G199V,G244V,G262delG,H179P,H193L,H193P,H214R,I162F,I232S,I251S,I255N,I255S,I255T,K132E,K132T,L145Q,L252P,M246K,P151H,P152S,P301fs*>41,P72R,R158H,R174W,R175C,R209fs*6,R213R,R248Q,R249G,R249S,R249W,R267L,R267Q,R280G,R282G,R283H,R290H,S215R,S303fs*>39,V157F,V157_R158delVR,V173A,V197M,V203L,V217G,V272E,V272L,V274D,V274L,Y220D,Y220H,Y220N,Y2 34*,Y234S,Y236*,Y236C |
| TP53_ENST00000455263 | A159P,A276D,A276P,C135W,C176G,C176Y,C229fs*10,C275G,C277Y,D208V,D281Y,E198*,E258G,E271*,F270L,G154V,G244D,G244R,H168L,H178D,H178P,H179N,I162S,I195N,I255F,K164*,L111R,L145R,M169fs*5,M237I,M246L,N235D,P151A,P152L,P152T,P152fs*18,P177_C182delPHHERC,P190L,P190S,P191delP,P250L,P278H,R156C,R158P,R175L,R248G,R273H,R273S,R282P,R283P,R290C,S166*,S215N,S240G,S241C,S241F,T125M,T155P,T253P,V147G,V218delV,V272G,Y163C,Y205C,Y205fs*4,Y220C,Y234D,Y236D,Y236 N |
| TP53_ENST00000545858 | C149Y,C83F,G151S,G152A,G152S,H86R,L101F,M144I,M144K,N146D,R120G,S122G,V12 3L,Y112F,Y141C,Y70N |
| TP63_ENST00000418709 | R379C |
| TSC1 | Q527*,R509* |
| TSC2 | V1711M |
| TSHR | A623S,A623V,D619G,D633E,D633H,D633Y,I486F,I486M,I568T,I630L,L512Q,L512R,L62 9F,M453T,T632I |
| U2AF1 | Q157P,Q157R,S34F,S34Y |
| VHL | C162*,C162F,C162R,C162W,C162Y,D121G,D121Y,E160*,E160K,E160fs*10,F148fs*11,F76del,F76fs*83,G114A,G114C,G114D,G114R,G114S,G144*,G144R,G144fs*14,G144fs*15,H115N,H115Y,I151N,I151S,I151T,L118P,L128H,L128P,L128R,L128fs*31,L153P,L158P,L158Q,L158R,L158V,L169P,L169fs*33,L184P,L184R,L85P,L89H,L89P,L89R,N78D,N78I,N78K,N78S,N78T,N78Y,N90I,N90fs*69,P81S,P81fs*50,P86H,P86L,P86S,P86T,R161*,R167G,R167Q,R167W,S111G,S111I,S111N,S111R,S111fs*48,S65*,S65L,S65P,S65T,S65W,S72P,S72fs*87,S80N,S80R,V130D,V130F,V130L,V155L,V155M,V155fs*4,V166D,V166F,V166G,V166I,V62fs*5,V74D,V74G,V74fs*85 |
| WT1 | A314fs*3,A314fs*4,A314fs*6,A314fs*67,A314fs*69,D396N,R312G,R312Q,R312fs*5,R 312fs*69,R312fs*72,R394L,R394P,R394Q,R394W,V303fs*14 |
| XPO1 | E571G,E571K,E571V,R749Q |
| XRCC1 | R194W |
